# Turning Negatives into Positives for Pet Trading and Keeping: A Review of Positive Lists

**DOI:** 10.3390/ani10122371

**Published:** 2020-12-10

**Authors:** Elaine Toland, Monica Bando, Michèle Hamers, Vanessa Cadenas, Rob Laidlaw, Albert Martínez-Silvestre, Paul van der Wielen

**Affiliations:** 1 Animal Protection Agency Foundation, Werks Central, 15–17 Middle Street, Brighton BN1 1AL, UK; 2 PETA Foundation, 501 Front Street, Norfolk, VA 23510, USA; monicab@petaf.org; 3 World Animal Protection, 90 Eglinton Ave East, Suite 960, Toronto, ON M4P 2Y3, Canada; MicheleHamers@worldanimalprotection.ca; 4 Animal Protection, Biodiversity and Natural Environment Section, Government of Catalonia, 43004 Tarragona, Spain; vanessa.cadenas@gencat.cat; 5 Zoocheck, 788 1/2 O’Connor Drive, Toronto, ON M4B 2S6, Canada; rob@zoocheck.com; 6 CRARC (Catalonian Reptiles and Amphibians Rescue Centre), 08783 Barcelona, Spain; crarc-masquefa@outlook.com; 7 AAP Animal Advocacy and Protection, Kemphaanpad 1, 1358 AC Almere, The Netherlands; Paul.van.der.Wielen@aap.nl

**Keywords:** exotic pet, pet, positive list, regulation, precautionary principle, wildlife trade

## Abstract

**Simple Summary:**

In regulating the trading and keeping of exotic pets, lawmakers seek to protect animal welfare, prevent species declines, and safeguard biodiversity. The public also requires protection from pet-related injuries and animal-to-human diseases. Most legislation concerning exotic pet trading and keeping involves restricting or banning problematic species, a practice known as “negative listing”. However, an alternative approach adopted by some governments permits only those species that meet certain scientifically proven criteria to be sold and kept as pets. Thus, governments may “positively list” only those species that are suitable to keep in domestic settings and that do not present a disproportionate risk to people or the environment. We reviewed international, national, and regional legislation in Europe, the United States, and Canada and found that largely unpublished and often inconsistent criteria are used for the development of negative and positive lists. We also conducted online surveys of governments, which received limited responses, although telephone interviews with governments either considering or developing positive lists revealed insights regarding their interest and motivation towards positive lists. We discuss key issues raised by civil servants including the perceived advantages of positive lists and challenges they anticipate in drawing up suitable lists of species. We compare functions of negative and positive lists and offer recommendations to governments concerning the development and implementation of positive lists.

**Abstract:**

The trading and keeping of exotic pets are associated with animal welfare, conservation, environmental protection, agricultural animal health, and public health concerns and present serious regulatory challenges to legislators and enforcers. Most legislation concerning exotic pet trading and keeping involves restricting or banning problematic species, a practice known as “negative listing”. However, an alternative approach adopted by some governments permits only the keeping of animals that meet certain scientifically proven criteria as suitable in respect of species, environmental, and public health and safety protections. We conducted an evaluation of positive lists for the regulation of pet trading and keeping within the context of the more prevalent system of restricting or prohibiting species via negative lists. Our examination of international, national, and regional regulations in Europe, the United States, and Canada found that criteria used for the development of both negative and positive lists were inconsistent or non-specific. Our online surveys of governments received limited responses, although telephone interviews with officials from governments either considering or developing positive lists provided useful insights into their attitudes and motivations towards adopting positive lists. We discuss key issues raised by civil servants including perceived advantages of positive lists and anticipated challenges when developing lists of suitable species. In addition, we compare functions of negative and positive lists, and recommend key principles that we hope will be helpful to governments concerning development and implementation of regulations based on positive lists.

## 1. Introduction

In both the scientific community and the popular media, there are calls for greater controls on exotic pet trading and keeping in response to the myriad of problems involved [1,2,3,4,5]. However, the form that these controls should take is undetermined. Post-millennium, governments have begun to establish a novel approach to regulating the exotic pet trade based on a precautionary principle, particularly in Europe and Canada [6,7]. Much of this regulation is still newly formed but has attracted the attention of other governments aiming to better control their national pet trading and keeping practices. In this article, we aim to assist such lawmakers by reviewing background information, exploring reported perceptions of government officials, discussing relevant considerations, and recommending key principles on which to base positive lists. For the purposes of this article, the term “exotic pet” will be broadly defined as a species that is non-native or non-domesticated.

### 1.1. Volume of Animals and Diversity of Species in the Pet Trade and for Private Keeping

Demand for exotic pets in the Western world is a major driver for the live wild animal trade and involves tens of millions of individual animals ranging from invertebrates to non-human primates [8]. The greatest portion of the exotic pet trade is comprised of ornamental fishes, amphibians, and reptiles—most of which are destined for Europe and the United States [8].

It is difficult to quantify the volume of wild animals traded as pets largely because poor record-keeping appears to be commonplace [9,10,11]. For example, the vast majority of exotic species are not listed in the appendices of the Convention on International Trade in Endangered Species of Wild Fauna and Flora (CITES), and data for non-CITES-listed species are sparse (i.e., not systematically recorded) [9,12,13,14]. However, Auliya et al. established that more than 20 million reptiles (CITES- and non-CITES-listed) were imported into European Union (EU) member states between 2004 and 2014 [10]. In the US, over one billion specimens, plus a further 977 million kilograms of wildlife, were imported between 2000 and 2013. One-third of shipments contained live animals, and the majority of these were for the pet industry [8]. Between 2007 and 2017, more than 23 million wild animals were imported into Canada for commercial or personal reasons [15]. The scale of illicit trade is manifestly unknown but is estimated to comprise at least one-quarter of the entire exotic pet trade [16]. Illegal trade in some species may even exceed documented legal trade [17]. Indications are that, overall, both international trade in exotic pets and exotic pet ownership are increasing [13,18,19,20,21], although some national trends (e.g., in the UK) suggest a decrease in recent years [22].

Around 85 million households in Europe own a domesticated or exotic pet [23], and there are a similar number of pet-owning households in the United States [24]. It is reported that over half of Canadians own pets [7]. Table 1 contains a breakdown of pet population by region.

At least 13,000 species (including >10,000 vertebrate species) are traded and kept as pets [26]. Species diversity in the pet trade is increasing [13] and such increase appears likely to continue [27]. For most traded species, knowledge of their natural biology is either absent or insufficient in terms of forming a reliable basis for husbandry guidance, or for assessing their potential threat to human health [26,28].

Newly described species are known to be rapidly exploited by the commercial pet trade to such a degree that conservationists are reluctant to publish their exact locations in case it harms the species’ survival [10,11,29,30,31,32]. In some cases, previously undescribed species were discovered that had already been well-established in the pet trade [33,34,35,36,37]. In 2017, a novel species of skink was on display and openly offered for sale at a German exotic pet market only three months after being discovered in the wild [38].

Although the exotic pet trade involves great diversity across all taxa, it tends to be dominated by a relatively small number of popular species [39,40], which indicates that great species diversity is not necessary for viable trade. The ability to produce unusual colour variations (“morphs”) of animals via artificial selection and inbreeding has increased the popularity of some species [41,42]. The trade is also driven by popular culture, and transient “fad” species can cause a surge in demand for certain animals [27,43].

Pet trading and keeping involves a raft of diverse and important considerations with intertwined ramifications. Accordingly, first, we provide background summaries to key areas associated with exotic pet trading and keeping; second, we provide results of our study of regulatory measures with a focus on positive lists, and third, we discuss various elements of and regulatory comparisons relevant to positive lists.

### 1.2. Animal Welfare and Consumer Issues

The commercial trades in domesticated and exotic animals as pets are associated with significant animal welfare concerns. Whereas, for trade in domesticated animals, key welfare problems relate to breeding practices, cross-border trade, and lack of owner knowledge [44,45,46], issues concerning exotic pet trading further extend to encompass the processes of wild capture as well as the fundamental unsuitability of many species for private keeping [18,26,28,47].

Animals are known to suffer at each stage of the exotic pet trade, and for many it begins with their extraction from the wild. Capture techniques for wild species can cause significant physical injury, stress, and death [13,48]. Once removed from their natural environment, forced confinement and close proximity to humans and other animals is also probably stressful [49]. Wild-captured animals are then stored prior to export in conditions that are dirty, severely overcrowded, and without access to food and water [50,51]. During transit, dehydration, starvation, injury, hypo- or hyperthermia, crushing, asphyxiation, disease, and stress contribute to high morbidity and, for some species, high mortality prior to export [52].

For those animals that survive capture, storage, and transport, poor animal welfare conditions are also documented at exotic pet wholesalers. An investigation of a major international pet wholesaler based in the US found that 80% of animals (invertebrates, amphibians, reptiles, and mammals) were diseased, injured or dead [53]. The associated mortality rate over a six-week period was 72% across all animal classes, and this figure was considered by trade sources to be “industry standard” for exotic pet wholesalers [53]. During other investigations of exotic pet wholesalers in Germany and the Netherlands, animals were observed suffering and dying under similar conditions [54,55].

Intensively managed captive-breeding operations for exotic pets are reported to involve poor welfare, because animals may be permanently held under conditions of deprivation involving restrictive and inappropriate enclosures, subjected to either stressful overcrowding or solitary confinement, and offered only minimal provisions of food and water [56,57,58]. Chronic or lifelong problems can result from some captive-breeding methods. For example, parrot chicks may be prematurely removed from parents and hand-reared, fed on poor diets, and kept under conditions that lead to malnutrition, skeletal deformity, and long-term behavioural problems [59,60]. Genetic disorders, commonly associated with domesticated companion animals, are also linked with exotic pet breeding. For example, artificial selection of reptiles is associated with the neurological disease “wobble syndrome” in royal python (*Python regius*) morphs, and can, amongst other problems, affect their ability to feed [58,61].

Animals are marketed to end consumers via physical or online pet shops or at temporary exotic pet markets (otherwise known as “shows” or “expositions”). Exotic pet markets supply wild-caught and captive-bred animals to breeders and retailers, as well as to the general public. Behavioural assessments of animals traded at such events have determined the prevalence of high levels of stress in animals [58,62]. Poor welfare conditions have also been observed in pet shops and at the premises of online traders that supply both pets and animals bred for reptile food [45]. Pet cages and equipment sold via pet shops or online are often inappropriate and advice on animal husbandry can be absent, incorrect or contradictory [28,63].

Once in private hands, exotic pets are typically housed in minimalistic and inadequate environments that lead to compromised welfare including spatial and behavioural deprivation, chronic stress, disease, and premature mortality [48,64,65]. In Germany, a survey conducted by specialist veterinarians of reptile keepers found major husbandry deficits: parameters were incorrect for humidity in 57% of cases, and for ambient air temperature in 43% of cases [66]. For some species, their fundamental natural behaviours are thwarted by poor captive conditions. Snakes are routinely confined to enclosures where they cannot stretch their bodies to full length [67,68]. The majority of pet rabbits and various small mammals are kept in severely restricted spaces that limit opportunities for physical activity and digging, provided insufficient diets that contribute to obesity and dental disease, and are singly housed despite being highly social [47,63].

Whereas traditional domesticated pets tend to achieve natural longevities, high premature mortality rates for exotic pets are common (at least 75% for reptiles and 70% for marine fishes within the first year of purchase) [69,70]. Although these mortality rates suggest a pattern of widespread neglect, a probable underlying cause is associated with the mis-selling of species that are fundamentally unsuited to captivity [47]. False and misleading marketing claims by the pet industry that many exotic animals are “easy to keep” or are “ideal starter pets” are common [26,28,71,72]. Research in Canada showed that 43% of first-time exotic pet owners purchased their animals on impulse [7]. Consumers are rarely advised at point of sale about the time, effort, space, financial resources, as well as expertise, needed to care for an exotic animal [26]. Furthermore, online animal-keeping forums along with books, care sheets, and websites produced by amateur or unqualified keepers and traders frequently understate the complexity of captive wild animal care, and incidentally promote what is now commonly referred to as “folklore husbandry” [26,67,71,72].

Recognising signs of stress or pain in exotic animals can be challenging for pet keepers, and failure to interpret—often subtle—behavioural indicators can mean that animals do not receive appropriate or timely veterinary or other expert care [18]. Where need for veterinary attention is identified, exotic pet owners may not seek, or have access to, the relatively small number of veterinarians who specialise in exotic species [18].

Often, poor welfare is exacerbated due to a lack of basic understanding about the complex needs of exotic species. However, many exotic species are inherently unsuited to private ownership as pets given their challenging captive husbandry requirements [47].

### 1.3. Public Health and Safety Risks

Wildlife trade has been identified as the most likely pathway for SARS-Cov-2, the virus responsible for the COVID-19 pandemic [73]. Tackling the legal and illegal trade in wild animals for pets or consumption has been identified as high priority in terms of preventing future disease outbreaks [1,74]. It is estimated that 75% of emerging infectious diseases are of wild animal origin [75].

Both domesticated and exotic pets present recognised risks to public health and safety as transmitters of zoonotic disease [9,75,76,77,78,79,80,81,82], and sources of antimicrobial resistance [83,84,85,86]. Human illnesses associated with domesticated species are generally within the familiar purview of ordinary veterinarians and medical doctors, thus facilitating relatively efficient diagnoses and treatments [87]. Within the last decade, there has been a significant research effort focused particularly on the health risks associated with exotic pet keeping [88]. However, the incidence and prevalence for most exotic pet-linked human infections are still unknown [89]. Much is known and understood regarding domesticated pet-linked human infection and treatment protocols, but exotic pet keeping can increase the risks of novel infections [86] and involve diseases that are more difficult to diagnose and treat [90].

Cases of zoonotic and other human infections associated with exotic pets are likely underreported because symptoms commonly resemble more regular illnesses. For example, gastrointestinal disorders linked to reptile-associated salmonellosis can be mistaken for typical food-poisoning, and flu-like disease linked to avian-associated psittacosis, both of which may originate from pets, can be wrongly interpreted [89]. Certain groups, such as the elderly, children under five years, pregnant woman, and the immunocompromised are more susceptible to pet-related infections [76].

The incidence and prevalence of dog bites vastly outnumber those of exotic pet-related injuries, even when adjusting for the considerably greater number of dogs present in homes [91,92]. However, efforts can be made to reduce dog bites by public education and responsible pet ownership, and it is conceivable that such measures will likely also reduce stress in dogs [93,94], whereas reducing the incidence and prevalence of exotic pet-related injuries is difficult to achieve given the unpredictability of aggressive and defensive behaviours in wild animals [92,95].

Physical injuries caused by exotic pets range from relatively innocuous bites and scratches from small animals to life-threatening attacks by larger animals such as carnivores, constrictor snakes or crocodilians [96]. For many primate and bird species, aggressive behaviour can be associated with reaching sexual maturity, or attempting to gain a position within a perceived social hierarchy [5,59]. Bites and stings from snakes, arachnids and aquatic animals such as sting rays and lionfish also account for some injuries. The most serious envenomations result from being in a close proximity to venomous snakes such as rattlesnakes, mambas, and cobras [97,98,99]. Certain types of injuries can result in secondary infections, and treatment may be protracted due to atypical pathogens [90,92].

### 1.4. Species Conservation and Illegal Trade

The uncontrolled commercialisation and demand for wild animals as pets is recognised as a significant and increasing driver of the global decline and extinction of species [10,12,100,101,102]. Collector demand is particularly high for rare species or those that have only recently been described [29,30,38,103,104]. Rarity of a species can enhance its desirability, which, in turn, stimulates higher prices; this is true of both legal and illegal trade [27,101]. High mortality rates during capture mean that the numbers of animals taken from the wild may be substantially underestimated [13]. For example, mortality rates prior to export may be as high as 100% for some birds in Senegal and Indonesia, 85% for some ornamental fishes in Hawaii and India, and 50% for some chameleons in Madagascar [105], and cumulatively these mortalities add to wastage of wildlife and compensatory collection.

Furthermore, there is increasing evidence of systematic trade in those species that are protected only by national legislation in their country of origin [32,34,106,107,108,109]. Such species, providing they are not protected by CITES (or in Europe by the EU Wildlife Trade Regulation 338/97) are being openly and legally imported, sold or kept in most countries, including the European Union [10,32,109,110]. This significant legal gap enables a pervasive and highly lucrative business comparable to the trade in CITES Appendix I and II species, but with no legal penalties for importers and traders [111]. The US, via its Lacey Act, is the only country that prohibits trade in species that are protected by the domestic legislation of other countries [32].

Complex legislation governing the exotic pet trade at both international and national levels can blur the line between legal and illegal trade. Just as an animal can be illegally captured in one country and legally sold in another, the same species can be legal or illegal depending on whether it has been wild-captured or captive-bred [21]. In addition, the trade in legally captive-bred species may contravene domestic legislation [62]. Criticism is often directed solely at illegal trade, but the systems that facilitate legal trade also enable illicit and unregulated trade to flourish. Quotas are exceeded, CITES paperwork is falsified [112], and smugglers may misreport the number of animals in a shipment, so that hundreds are transported in overcrowded conditions that promote morbidity and mortality [113]. There are also numerous reports of illegally wild-caught reptiles being laundered through wildlife breeding farms and wrongly declared as captive-bred [114,115].

### 1.5. Invasive Alien Species

The trade and keeping of exotic pets represent a major pathway for the introduction of invasive alien species (IAS), which can be detrimental to local and regional biodiversity [21,116]. An IAS can threaten native wildlife through such means as predation, competition, hybridisation or disease introduction [117,118]. A notable example is the Burmese python (*Python molurus bivittatus*) which, following its popularity in the pet trade, has become established in south Florida as an apex predator and causes declines in native wild animal populations [119]. The Burmese python is only one of at least 140 non-native amphibians and reptiles that had been introduced into Florida by 2010, almost 85% of which had arrived via the pet trade [120,121,122,123]. By 2015, an additional 38 invasive alien species had been recorded [124]. Overall, the pet industry is responsible for most non-native populations of amphibians and reptiles worldwide [21]. The most commonly traded amphibian and reptile species in the US are American bullfrogs (*Lithobates catesbeianus*) and slider turtles (*Trachemys* sp.), and both are amongst the world’s most invasive species [39].

The issue of IAS is most commonly associated with the exotic rather than domesticated pet trade. The process of artificial selection over thousands of years can result in the loss of physical and behavioural attributes that severely limit the likelihood of most domesticated animals being able to thrive in the wild [125]. Exceptions to this are feral dog populations, which can threaten human health and safety, damage crops, and kill livestock [126] and free-ranging domesticated cats, whether owned or feral, which can present a significant threat to endemic wildlife through predation [127].

It has been reported that novel non-native populations of amphibians, reptiles, birds, and mammals in the EU are attributable to released or escaped pets [128]. Indeed, the pet trade is the primary source for released and escaped non-native birds in Europe [129]. The increase in non-native fish species in freshwater bodies and marine coastal waters of North America and the EU is also attributed to the growth in ornamental fish-keeping [21].

It is not fully understood why exotic pet owners release their pets, but it may be a result of their struggle to meet the care requirements, particularly of exotic animals [18]. Nevertheless, it is estimated that a ten-year time lag exists between a species becoming popular in trade and it subsequently being recorded as introduced in the wild [21]. The economic impact of IAS is substantial. The EU, for example, spends at least EUR 12 billion and probably over EUR 20 billion per year on the damage caused by IAS as well as prevention, control, and mitigation measures [130].

### 1.6. Disease Threats to Wildlife and Agriculture

Disease transmission via the global wildlife (exotic pet) trade is responsible for significant threats to native wildlife and agriculture [16,131,132]. For example, psittacine beak and feather disease is highly infectious, and all psittaciformes are susceptible to infection. The disease is found in both wild and captive birds and the global spread has been linked to the large international trade in pet parrots [133,134]. At least 33 countries have reported the disease in captive parrots [133].

The threat to wildlife from emergent diseases associated with amphibians and reptiles traded as exotic pets has been widely described. For example, highly infectious chytrid fungus has been responsible for severe population declines of endemic amphibians worldwide [135,136,137] and a high prevalence of diseases such as herpesvirus or *Mycoplasma* spp. has been detected in invasive turtle populations [138]. Several tick species have been reported on all groups of exotic reptiles legally imported to European countries [139,140,141].

The raft of emerging and re-emerging diseases affecting agriculture over the past 30 years have cost world economies many billions of dollars [142], and many disease outbreaks are directly related to the trade in wildlife. Arthropod-borne pathogens can be carried over long distances by reptiles in commercial trade. African tortoises in the pet trade have been associated with the tick-borne pathogen *Ehrlichia ruminantium*, which is responsible for the often fatal heartwater disease in cattle [53]. Recent reports show that tortoises act as hosts for ticks harbouring the virus that causes Crimean–Congo haemorrhagic fever—a virulent pathogen that can be transmitted to both livestock and humans [143]. Highly pathogenic avian influenza (H591) and Newcastle’s disease (Paramyxoviridae) are highly significant and now endemic threats to agricultural production of poultry, with flock mortalities of up to 100% [144]. The avian influenza H5N1 virus was isolated in mountain hawk eagles illegally shipped from Thailand to Belgium, and in a consignment of pet birds transported from Asia to the UK. Newcastle’s disease entered Europe via a shipment of parrots and finches imported from Pakistan [16].

### 1.7. Our Study

Efforts to control the exotic pet trade and mitigate its effects typically involve implementing bans or restrictions on certain species, which conforms to reactive regulatory systems otherwise known as “negative lists”. However, in recent years, several governments around the world have adopted the precautionary principle, which conforms to proactive regulatory systems otherwise known as “positive lists” when managing the trade and keeping of exotic pets. Thus, governments using positive lists permit in trade or keeping only those species that meet certain safety criteria, so that any potential adverse effects are minimised.

A substantial evidence base confirms persistent multifactorial problems associated with exotic pet trading and keeping under reactive controls, thus current and predominantly negative list-based, regulatory systems are manifestly failing to protect biodiversity; conserve wild animal populations; curb wildlife illegal trade; safeguard human health and animal health and welfare. We aimed to conduct an evaluation of positive lists for pet trading and keeping, and to assess the level of governmental interest in this type of system. We examined existing criteria used for negative and positive lists in Europe, the US, and Canada; surveyed governments regarding the use of positive lists; discussed the precautionary principle as it applies to the pet trade; as well as set out recommendations for implementing positive lists.

We define negative lists systems as regulation that restricts or prohibits trade and/or keeping of certain species. Species are banned typically on the grounds that they are: unsuitable to keep in the home in terms of animal welfare; or proportionately harmful in terms of human health and safety; or unsustainable in terms of relevant conservation status; or inconsistent with environmental preservation. Certain species may be permitted for selling and private keeping with a special permit (for example, by those who can demonstrate that they have specialist facilities or expertise).

We define positive list systems as regulation that permits the trade and/or private ownership of only those species that are determined as: suitable to keep in the home in terms of animal welfare; or proportionately benign in terms of human health and safety; or sustainable in terms of relevant conservation status; or consistent with environmental preservation. All other species are by default prohibited from being sold or kept privately or may only be sold or kept under special permit (e.g., by those who can demonstrate that they have specialist facilities or expertise).

Negative and positive lists may include entire classes of animals or particular breeds of a single species, and any taxonomic category in between. For example, a negative list may include dog breeds that are considered dangerous [145]. Lists can also encompass groups of animals according to their conservation status or provenance—for example, a negative list of endangered or wild-caught animals [146]. In the context of selling pets, negative and positive lists can pertain to specific activities, such as importing, exporting, or selling through pet shops, or can even apply to particular internet platforms. The scope of this paper encompasses legislation that refers specifically to selling and/or keeping pets, and/or broadly impacts pet trading and keeping, in particular in the context of species lists.

## 2. Materials and Methods

### 2.1. Desk Research

For the purposes of analysis, we divided regulation into two main areas. Firstly, we conducted online searches, and compiled and summarised laws that apply internationally, including at the European Union level, and federally in the US and Canada. Secondly, we collated legislation that comprises negative and positive lists for pet trading and keeping that applies federally in European countries and at the state/province level in the US and Canada, at which level statutes are broadly equivalent in Europe and North America.

Some Canadian municipalities already known to have positive lists were also included in the study. Europe was defined as including members of the European Single Market [147]. Because animal welfare regulation is devolved in the UK between England, Wales, Scotland, and Northern Ireland, it was separated into these four administrations. For the same reason, Belgium was divided into the regions of Brussels, Flanders, and Wallonia. Germany, Austria, and Spain also have specific animal welfare legislation at the regional level but have concurrent national legislation and so were not divided into separate entities. Where relevant information was available, we compared criteria for inclusion of species on negative and positive lists and explored factors that motivated governments to permit or prohibit certain species. At both international and national levels, positive lists in the form of exemptions were not included if they were implemented primarily for the purposes of hunting.

### 2.2. Surveys

#### 2.2.1. Online Surveys

We conducted two online surveys via research software, SurveyMonkey, targeting all European governments, states in the US, and Canadian provinces and territories. Questionnaire A (Supplementary File) was sent to governments without positive lists to assess their level of interest in positive lists. Questionnaire B (Supplementary File) was sent to governments with positive lists (including Canadian municipalities and towns known by our team to have positive lists). At the preparatory stage, we contacted governments to request email addresses for relevant civil servants that oversaw regulation specifically pertaining to exotic pet trading and keeping. Where it was not possible, for a number of reasons, to obtain email addresses for relevant officials, we were directed by government switchboard staff to use email addresses for relevant departments, such as for agriculture, environment, wildlife or even to use generic email addresses for public enquiries. Questionnaire A (Supplementary File) was sent to multiple contacts per government where needed. However, we were only able to send Questionnaire B (Supplementary File) to one contact in each government, because responses were anonymised, and it was necessary to guard against processing more than one completed questionnaire per government. For the same reason, the software did not permit recipients of Questionnaire B to forward the email to colleagues.

Informed consent was sought on the online landing page and guarantees of confidentiality were provided. Information was sought regarding the common criteria used to develop positive lists; the challenges encountered in formulating the lists; the effectiveness of positive lists and the nature of the public response to positive lists (see Supplementary File for Questionnaires A and B). Questionnaires were pre-tested at the design and distribution stages. The survey took place between 7 August 2020 and 9 September 2020, and questionnaires were issued in English. Non-respondents were reminded up to five times to participate in the survey. Data from Questionnaire A were aggregated and Questionnaire B was anonymised.

#### 2.2.2. Telephone Interviews

Short telephone interviews (Supplementary file) were offered to Questionnaire A respondents who indicated that they would consider, or were considering, a positive list or were already in the process of developing a positive list. The purpose of the telephone interviews was to gauge early feedback from those countries considering or developing positive lists.

Two project team members conducted the semi-structured interviews. Interviewees were asked to describe: their departmental role with regard to regulating exotic pet trading and keeping; what prompted the government’s interest in positive lists; what current problems were being observed in relation to exotic pet trading and keeping; how they would envisage positive lists addressing those problems; what challenges they would anticipate in introducing positive lists (see Supplementary file for Telephone Interview Questions).

Informed consent was obtained at the time of the interview and transcripts were anonymised. Subject patterns, or codes, were devised from a thorough reading of the transcripts using an inductive approach and based on an objective assessment of subjects most frequently mentioned. NVivo (NVivo release 1.3, QSR International) qualitative data analysis software was then used to assist with the coding and thematic breakdown of the transcribed conversations. Points of commonality were grouped together until major themes emerged.

## 3. Results

### 3.1. International and Federal (US and Canada) Legislation

At the international level, including the European Union, and at the federal level in the US and Canada, all regulation pertaining to pet trading and keeping is based around a negative list system. Notwithstanding this fact, the Wild Bird Conservation Act of the US contains exemptions that meet the definition of a positive list (see Table 2).

The Bern Convention and the EU Habitats Directive also contain lists of species for which exploitation may be permitted. However, species listed in Appendix III of the Bern Convention and Annex V(a) of the EU Habitats Directive’s may be subject to different management measures within the various signatory countries or EU states. Therefore, these regulations were not sufficiently delineated to be characterised as positive lists. Similar reasoning was applied to The Migratory Bird Treaty Act of the United States, which lists non-native bird species to which the Act does not apply, but these may be protected by other international agreements and statues. Likewise, under the Migratory Birds Convention Act, a list of bird families that are not protected by the Act may be protected under provincial or territorial legislation, or by other federal conventions, so was not classed as a positive list.

### 3.2. Legislation in European Countries

There is no EU Regulation specifically concerned with the welfare of pets or their risks to public safety, thus each member state applies its own national legislation in this regard [6]. Most European countries have some form of restrictive regulation on the keeping of exotic pets and this is mostly in the form of negative lists [6,148].

To date, six European countries, Belgium, Luxembourg, Norway, The Netherlands, Malta, and Croatia have introduced or legally enshrined the principle of positive lists. The latter three of these also have negative lists [6] (see Table 3). The remaining countries all have negative lists, which generally incorporate restrictions pertaining to CITES, the Invasive Alien Species Regulation and the Wild Bird and Habitats Directives. Most European countries also restrict, via prohibitions or licensing/permit schemes, the trade and/or keeping of dangerous species. Other negative lists are implemented for protection of native fauna or animal welfare [6,148].

The first European positive list was introduced in Belgium in 2009 for mammals and contains 42 species. The Belgian Government originally introduced the same list in 2001 but it was legally challenged in 2007 on the grounds that it hindered trade between EU member states. In June 2008, the European Court of Justice ruled that the Belgian positive list was not in violation of EU free trade regulations as long as it was based on objective, non-discriminatory criteria and that a procedure was in place for parties to request the inclusion of species on the list (Andibel ruling) [149]. The Belgian Royal Decree of 2009 therefore reflects this ruling [150].

The assessment criteria employed for inclusion on the Belgian mammal list were that a species: must be easy to keep in terms of its basic physiological, ethological, and ecological needs; must not present an overt risk of becoming invasive in the natural environment; must not pose a disproportionate risk to human health; must have reliable husbandry guidance available. Where evidence is inconclusive, the benefit of the doubt must be given to the animal so that it is not listed [150].

In Belgium, competence for animal welfare was recently devolved to the three regions (Brussels, Wallonia, and Flanders). Each region has carried forward the positive list for mammals, subject to some slight amendments, into their respective legislation. Flanders has also implemented a positive list for reptiles [6], and Wallonia is currently in the process of formulating positive lists for reptiles and birds [151]. In the Brussels region, a positive list for reptiles is going through parliamentary stages.

Luxembourg’s positive list of 30 mammals closely resembles that of the Belgian list and was based on the criteria that animals must be able to be properly cared for and must not pose a risk to public safety. Alongside the mammal list, the Luxembourgian Government uses minimal restrictive criteria to control the keeping of other vertebrate classes as well as invertebrates. For example, non-venomous reptiles, amphibians and invertebrates, along with snakes, lizards, and crocodilians below a certain size are permitted [152].

Malta has a positive list for mammals, birds, reptiles, ornamental fishes, and other aquatic organisms. However, the list only applies to the sale of these animals through pet shops and does not apply to pet keeping. In addition, there is a separate list of bird species that are permitted to be sold through markets [153]. The positive list in Croatia is not specific to pet trading and keeping, but falls under its Nature Protection Act, encompassing forestry and hunting purposes and contains a list of plant species as well as animals [154].

In 2017, the Norwegian Government introduced a positive list of 19 reptile species [6] with the stated aim of better safeguarding animal welfare. This replaced a ban on reptile keeping that had been in force since 1976. The regulations oblige reptile importers, other traders, and keepers to have written documentation to demonstrate that their animals are second generation captive-bred [155]. The trade and keeping of amphibians as pets were also banned in 1976 and remain prohibited [155]. Norway also has a positive list for mammals, which restricts the sale and private ownership of mammals to those species that are traditionally kept in the home.

In the Netherlands, a positive list for mammals was developed, based on the criteria of animal welfare, public safety, and IAS risk. The list was enacted in 2015 but was repealed in 2017 following successful legal challenge on the grounds that it had not been prepared with due diligence. At the time of writing, a new list is being formulated following a different scientific methodology [6].

### 3.3. Legislation in States of the US

Exotic pet ownership in the US is not governed by any single federal law but is legislated predominantly at state and local municipal levels [156,157]. Legislation varies widely and is mainly in the form of negative lists. Regulation concerning exotic pet keeping has proliferated in recent years [158]. At the time of writing, online research identified 20 states of the US with bans on private possession of animals considered to be dangerous. Partial bans, allowing the private ownership of some exotic species, including small primates, operate in 13 states. Permits or licenses are required to own certain exotic species in 14 states, and in five states there are no regulations for exotic pet ownership [159].

In some states, local counties and municipalities may be empowered to introduce more stringent legislation than that which is in force at the state level [157]. For instance, hundreds of county laws and ordinances in Wisconsin prohibit the keeping of certain reptiles [160]. Regulation at the local level tends to be in the form of prohibitions, as counties and municipalities lack the necessary resources and infrastructure to administer permit or licensing regimes [158].

US lawmakers regulate exotic pet ownership for three main reasons. The first reason is public safety, in order to protect the public against escaped dangerous animals, and also to protect exotic pet owners. The second reason to introduce regulation is to mitigate zoonotic disease risks. The third reason is for animal welfare considerations [156].

Widespread media coverage of human injuries or fatalities resulting from exotic pet keeping may be a reason why public safety issues prompt more new regulation than disease risks [158]. For example, in Zanesville, Ohio in 2011, a suicidal exotic animal keeper released his 56 pets including big cats, wolves, primates, and bears before most were shot and killed by the authorities whilst wandering the neighbourhood. This tragic event prompted Virginia, Arizona, Missouri, Tennessee and West Virginia to introduce stricter regulation on exotic animal ownership [156]. Prior to the incident, Ohio had few restrictions on exotic pet ownership [161], but soon afterwards developed a long list of banned dangerous wild animals [162].

Species considered to be “dangerous”, “wild” or “deleterious” vary considerably between states. For example, some states consider primates, crocodiles, and anacondas to be dangerous, whilst other states may limit “dangerous” species to select large carnivores such as bears and some large cats. Yet other states, such as Nevada, have no restrictions on owning large cats such as tigers, primates, or elephants [163].

Some states prioritise the threats that exotic species present to the environment, livestock, and agriculture. For example, Idaho requires a permit to keep any “deleterious exotic animal” in order to safeguard the environment, livestock, agriculture, or wildlife of the state [164]. In response to the invasion of Burmese pythons in the Florida Everglades, the State of Florida introduced a permit system to keep pythons that required owners to pay an annual permit fee for each python and to have each animal microchipped [156].

At the time of writing, positive lists were identified in 21 states (see Table 3) and largely exist alongside established negative lists. Most of these lists are for species deemed to be unregulated “domestics” or otherwise exempt from some or all permitting regulations. Positive lists varied widely and in some states such as Tennessee, Wyoming, Florida, and Massachusetts, it was unclear on the face of the regulation whether species included on the positive list of one state department may fall under the different restrictive regulations of another department. Some states grouped import, possession, and other activities together with respect to negative or positive lists whilst in other states, regulation of different activities was separated between different divisions or departments. Thus, different restrictive or permissive requirements could apply to the same species depending on the activity.

The criteria used to formulate positive lists are often not readily discernible to the general public via online searches. From lists of prohibited species, it is possible to deduce that exempted species are typically those deemed not dangerous to humans or detrimental to the environment, agriculture or the state’s wildlife. However, while some states exclude invasive species from their positive lists, other states may include potentially invasive exotic species, but stipulate that these species must not be released into the wild and/or are housed in a manner that prevents their escape. Some states including Arkansas and Kentucky have positive lists of native species that can be collected in limited numbers as part of wider measures intended to prevent overexploitation of native fauna.

### 3.4. Legislation in Canadian Provinces/Territories and Municipalities

The keeping of wild animals as pets in Canada is largely regulated by provinces/territories and municipalities rather than at federal level. Most jurisdictions operate negative lists. However, in the late 1980s and early 1990s, four Canadian provinces and one territory adopted regulations that departed from the traditional negative list system. These regulatory frameworks range from standalone positive lists, as found in New Brunswick and Saskatchewan, to a combination of negative and positive lists, as in Newfoundland and Labrador, and Nunavut [7]. The province of Nova Scotia regulates pet keeping through both negative and positive lists and has a permit system for those species not included on either list, for which applications are considered on a case-by-case basis [165]. However, these policies are not enshrined in any regulations.

There are 3573 municipalities in Canada [166], of which 1586 are subject to the provincial or territorial lists of Alberta, New Brunswick, Newfoundland and Labrador, Nova Scotia, Saskatchewan, and Nunavut. Added to these are the municipalities of Ontario and Quebec that have implemented positive lists through their own bylaws (see Table 3). This means that a total of approximately 45% of Canadian municipalities are either subject to or apply positive lists. Based on our information, only municipalities in Quebec and Ontario have implemented positive lists and most of these were introduced after 2010. At the time of writing, Ontario has no provincial legislation on exotic pet ownership. Therefore, municipalities within Ontario have to determine which animals can or cannot be kept within their jurisdiction. All Canadian municipalities are empowered to adopt greater controls than those that exist at the provincial level. This situation is the case in the province of Quebec, which has a negative list, but several of its municipalities have implemented positive lists.

There is an apparent lack of consistency between the legislation of the various jurisdictions, which may stem from the fact that criteria on which negative and positive lists are based are often unclear. In some cases, a lack of taxonomical knowledge is evident. For example, some positive lists include an individual species alongside the genus to which it belongs, thereby introducing unnecessary duplication.

Canadian positive lists often include entire (taxonomic) families rather than individual species. As a result, most invertebrate, amphibian, reptile, and parrot species are permitted to be kept as pets. In some cases, the entire reptile class is listed, with the exclusion of those that are venomous or above a certain size. Such blanket listing results in thousands of reptile species being designated as suitable pets.

Public safety in Canada has historically been the main driver for the review or implementation of new exotic pet legislation, as is the case in the US. For example, an incident in 2007 that involved a woman being mauled to death by a pet Siberian tiger (*Panthera tigris altaica*) [167] prompted British Columbia to implement the Controlled Alien Species Act. The Act includes more than 1000 species on a negative list based on the threat they pose to people, property, wildlife, and the natural environment [168].

A similar response was elicited from the New Brunswick Government after two young boys were killed by a pet African rock python (*Python sebae*) in 2013. The incident led to New Brunswick implementing the Exotic Animals Act in 2017, which prompted a review of the existing positive list [169]. The Act is notable in terms of the principles on which it is based as, unlike many other jurisdictions, it extends beyond safeguarding public health and safety. The stated purpose of the legislation is also to protect native biodiversity and to take into account the conservation status of exotic animals as well as their welfare needs [170]. Nova Scotia’s Wildlife Regulations also employ criteria that benefit animal welfare, local biodiversity, and species conservation, for example, by aiming to ensure that captive wildlife is disease free, properly housed and not released into the environment [165].

The regulatory language used to describe which animals can or cannot be kept as pets is often vague and confusing. For instance, several municipalities use double negatives, such as: “it is prohibited to be in possession of an animal that does not appear on this positive list”. The positive list of one municipality allows birds “except those which are prohibited”. However, the bylaw fails to specify which birds are prohibited. Another municipality prohibits the possession of an animal that is not domestic but provides no definition of “domestic”. Some municipal positive lists are not prescriptive and only include examples of species that fit a broad definition of “companion animal”.

### 3.5. Survey Results

#### 3.5.1. Online Survey

Questionnaire A (Supplementary File) was sent to a total of 58 governments that, to our knowledge, do not already have positive lists. This list comprised all of the European countries (*n* = 26), states of the US (*n* = 24), and Canadian provinces/territories (*n* = 8) that are not included in Table 3. Of these countries, states and provinces/territories, eleven (19%) governments responded. All the responding governments came from Europe: five (9%) confirmed that they did not have a positive list and were not developing or considering a positive list; three (5%) indicated that they were considering a positive list; three (5%) were in the process of developing a positive list. One respondent advised us separately that they were unable to answer the final question as to whether they would consider a positive list, so did not complete it.

The non-response bias for North America may have been due to problems encountered in targeting the survey at appropriate officials. Sourcing correct email addresses was frustrated by various complications. For example, in some states of the US, there is a shared responsibility between different agencies for implementing and enforcing regulation concerning exotic pet keeping. Departments that issue permits for possession of exotic/wildlife species may be different to those responsible for enforcing regulations pertaining to possession of exotic/wildlife species. In Canadian municipalities and some states of the US, we were unable to access email addresses for relevant officials or departments and could only obtain generic email addresses used for public enquiries. In addition, surveys were sent out during the COVID-19 pandemic and some U.S. agency offices were closed and/or staff were furloughed.

Spam filtration and firewall issues may also have reduced the overall response rate. We sent test emails to eight recipients: of these, two emails did not arrive, were not recorded on the systems as “bounced” and were not found in spam folders so therefore must have been blocked by firewall filters. These technological barriers are common problems with online surveys [171].

We speculate that as 50% of European respondents confirmed their interest or engagement on the positive list issue, this would explain their motivation for responding. Five governments that were considering positive lists agreed to participate in follow up telephone interviews.

Questionnaire B (Supplementary File) was sent to the 52 governments listed in Table 3 that met our definition of having a positive list. This included European countries (*n* = 8), states in the US (*n* = 21), Canadian provinces/territories (*n* = 6), and Canadian municipalities and towns (*n* = 17). A total of six (11%) responses, including one partial response, was received. Although responses to this questionnaire were anonymised, answers revealed unequivocally that at least two originated from Europe and at least two from North America (at least one of which was from Canada).

Of the five complete responses to Questionnaire B, all respondents said that they “agreed” that positive lists, as a means to regulate the exotic pet trade, were an improvement on the previous system and four respondents stated that overall, the public were supportive of positive lists. However, given the low response rate, we were unable to draw meaningful conclusions from the data received, including with regard to the common criteria used to develop positive lists and the challenges encountered in formulating the lists.

#### 3.5.2. Data from Telephone Interviews

Semi-structured interviews were conducted with five civil servants (from a pool of six online survey respondents who confirmed their interest in positive lists). All interviewees held senior positions within departments of agriculture, wildlife, animal welfare or nature conservation as a head of department, undersecretary, policy manager or senior specialist. Three of the interviewees had stated that their governments were considering positive lists, and two were in the process of developing positive lists. Interviews were 20–40 min in duration. Six major themes were identified from the interview transcripts:

- Theme 1: Perception that positive lists are a sound principle
  All interview subjects endorsed the principle of positive lists—a predictable response given that all had been selected for interview on the basis of their interest in positive lists. References were made to positive lists as being a “good idea in principle” or a “better approach”. Two interviewees referred to the views of their colleagues along with their own: “it seems to have been accepted as the logical way forward” and “my colleagues were receptive to it overall. Now it is agreed that we should try to get positive lists through”. All interviewees pointed to perceived advantages of positive lists, including: “the legislation involved would probably be easier in terms of keeping it up to date” and “illegal trade will continue but it will be easier to control”.
  Two of the group, whilst acknowledging theoretical advantages of positive lists, had no immediate plans to pursue their adoption. One had concerns about the effect on amphibian and reptile keepers: “a positive list wouldn’t allow them to keep animals that they almost have a professional interest in”. Another subject saw positive lists as a possible long-term objective: “increasingly strict regulation is justified and perhaps a move towards the positive list in the future”.
- Theme 2: Perception that positive lists would offer legal clarity
  Four interview subjects raised concerns about current regulation that is inadequate, unclear, or difficult to enforce or oversee. One interviewee advised that they had no defined legislation on exotic pet ownership and that internet pet sales were unregulated. Positive lists were seen as “[meeting a] need to fill a gap in the legislation” as “[exotic pet ownership] is not regulated and not under any control”. Another interviewee explained that enforcement authorities had complained that legislation was unclear and that it was difficult for members of the public to know whether they could import or keep exotic animals. It was seen that positive lists would “clarify the situation for the citizens”. This point was echoed by another interviewee who stated that a positive list would mean greater clarity for the general public in terms of what they can or cannot keep. This interviewee went further: “at present, the identification of different species from all countries of the world represents a great problem at the control points since it requires specialised knowledge”. A fourth interviewee stated that “we have different legislation for animal welfare, species protection, public health. It would be helpful to have one statute that addresses all of these related issues. We can’t be specialists on everything. As bureaucrats we have to be familiar with all these pieces of legislation”.
- Theme 3: Perception that positive lists would benefit animal welfare
  Four interview subjects referred to perceived benefits of positive lists to animal welfare: “it would ban the keeping of unsuitable species” and “there would be more species kept with no welfare issues”. Interviewees referred to current problems that could be addressed by positive lists: “there are certain exotic animals that require specialist care. These could end up in the hands of anyone” (unless covered by relevant regulation) and “for our animal welfare checks we are having to do research or seek information from zoos. Violations are hard to prove”. One interviewee stated that: “we would like to prescribe which species are allowed for an ordinary person with ordinary knowledge, and what conditions need to be met”.
- Theme 4: Acknowledgement that there would likely be opposition from trade
  Four interview subjects commented on their anticipated opposition to positive lists from exotic pet traders and hobbyists with varying degrees of concern. One interviewee stated: “the hobby organisations have reservations about it, which is understandable” In referring to a past consultation, one interviewee stated: “we had quite a backlash from the hobbyist community a few years ago”. Another interviewee affirmed that: “due to economic interests, the sectors involved will offer resistance and will oppose its application because what they are interested in is a market that is as free as possible for fauna”.
- Theme 5: Perception that positive lists may prevent exotic pet trade from getting out of control
  Three interview subjects described their domestic exotic pet trade as “small” or “not a major problem”. Of these, one expressed concern that demand for exotic pets may increase in line with improved standards of living, and another confirmed that exotic pet keeping was already becoming more popular, adding that “we don’t want to become a country where people keep animals because it’s trendy”. In this context, positive lists were seen as an intervention with potential to bring the exotic pet trade under control. Two of these subjects described issues with animal imports from EU countries, and a lot of animals coming from exotic pet markets abroad. One interviewee explained that authorities were having to be reactive, as keepers and dealers could seek retrospective permission after animals had already been imported, whilst also acknowledging that imports brought to their attention may be the “tip of the iceberg”.
- Theme 6: Concern regarding species selection for positive lists
  Three out of the five interview subjects spoke about being daunted by the prospect of drawing up a positive list, describing it as “the biggest challenge”; “we have no expertise to make the list”; “how do you decide what animals go on the positive list?”. Two of the three interviewees stated that they would take a stepwise approach and start with a mammal list but anticipated greater difficulty in formulating lists for other taxonomic classes. One interviewee acknowledged that geographical climate considerations meant that they would not be able to simply replicate another country’s positive list.

The above themes were delineated by identifying the common subject areas spoken about at most length. However, by this method, emphasis on certain issues was not taken into account. For example, the question of how to introduce schemes for licensing or registration and identification of species alongside positive lists was a major concern for two interviewees. Compliance with intra-EU trade regulations was also a key concern for three interviewees. The subject of international information sharing was frequently raised to explain how governments first became aware of the concept of positive lists and also in terms of expressing a need to learn from the experience of other governments.

Official roles of interviewees may have determined the subject areas on which they focused. One civil servant who had conducted focused work on invasive alien species issues described the exotic pet trade as causing an “economic problem of the first magnitude”. There was only one mention of public health risks associated with exotic pet trade and ownership, which may have been due to the fact that most interviewees worked in the areas of animal welfare and conservation.

## 4. Discussion

An outline of the precautionary principle and positive lists is presented below, followed by the main findings from the legislative review and a summary of species assessment tools. A comparison of negative versus positive lists is set out and recommendations are made regarding key principles to adhere to when developing and implementing positive lists.

### 4.1. The Precautionary Principle and Positive Lists

The precautionary principle is enshrined in national and international legislation [172,173,174] and can be applied generally by policymakers. The purpose of the precautionary principle (or precautionary approach) is to guide decision-making in the event of scientific uncertainty and, in effect, would mean that there would be no trading and keeping of animals as pets until all elements had been proven safe. Positive lists for pet trading and keeping apply the precautionary principle to species that cannot be permitted due to insufficient evidence.

The precautionary principle and positive list systems are commonly and normally applied to suitability criteria across diverse industries and other sectors, for example: products (including in respect of food additives, drugs, electrical goods, buildings and other structures, terrestrial vehicles and marine vessels, and others); professional practices and procedures (including in respect of medicine, surgery, dentistry, veterinary medicine, engineering, pilotage, law, and others); public activities (including in respect of vehicle driving licenses, passports, travel visas, and others). All these examples involve items or people first establishing their suitability for inclusion on (positive) lists that permit each activity. Accordingly, both the use and acceptance of the precautionary principle and positive lists are normalised within societies globally and must be impartially demonstrated and confirmed safe prior to being realised.

In this regard, the pet trade is atypical, and this discrepancy has been clearly illustrated via a comparison of a soft toy with a live turtle. The soft toy carries a label to show that it has been safety tested, and so is fire-resistant, machine-washable, and does not present a choking hazard. Whereas, a live turtle can bite, scratch, transmit disease via direct or indirect contact, and is sold with little or no objective husbandry information, and without reliable safety assessment [26].

The theoretical and practical benefits of positive lists for pet trading and keeping were acknowledged by civil servants during the interviews. There is support from within the academic community for positive lists as a means of aiding species conservation and protecting biodiversity [12,32,175,176,177,178], and from within the veterinary community for safeguarding animal welfare and preventing zoonoses and other infections [179,180,181,182].

### 4.2. Legislative Review

Our compilation of relevant international, national, and local legislation reveals a snapshot of the current regulatory landscape in which some positive lists have emerged but is still overwhelmingly dominated by negative list-based regulations. There may be a number of factors that explain the predominance of negative lists. In Western democracies, the principle of liberty and private property undergirds legal systems. There was, and remains, a general reluctance to interfere with people’s civil liberties and their right to own property including animals—which are still considered to be property in law. Greater restrictions on exotic pet ownership in the form of positive lists may be interpreted as infringing on the freedom and rights of citizens.

In North America, early legislation to control exotic pet keeping was almost exclusively for the purposes of addressing safety concerns and then later, and to a lesser extent, to tackle public nuisance problems. Historically, some Canadian Governments, particularly at the municipal level, did not have the clear legal authority to regulate exotic animal keeping except for public safety reasons, but the situation has gradually evolved. For example, in Ontario, the Municipal Act (which governs how municipalities are operated) was revised in the early 2000s resulting in an expanded capacity for municipalities to address animal control issues. Now, animal welfare, local environmental protection, and other issues can be taken into account.

Along with expanded legal capacities in some jurisdictions, the last 20 years has seen a change in public knowledge and sensibilities around animal welfare. Increased public concern, coupled with greater capacity for public oversight, via camera phones, and social media, has politicised the general public, and to a certain extent this has driven public policy.

It is perhaps understandable that a default response of politicians and governments to a specific problem may be to simply ban it. For example, a typical response to a serious injury or fatality inflicted by an exotic pet would be to prohibit that and other similar species from trade and private possession. These measures would seem defensible and proportionate, whereas a more proactive approach would have removed these risks to public safety. It may also be the case that negative list-based restrictions may be considered to be adequate in certain jurisdictions where exotic pet trading and keeping are relatively uncommon.

At the local level, resources are often a key consideration when planning control measures, as is the case in the US where local governments typically prohibit certain species from ownership because bans are perceived to be less costly than permitting regimes. It was also apparent from the telephone interviews that some civil servants were alerted to the concept of positive lists from learning of their implementation in other countries and may not otherwise have considered this approach.

Of course, legislation is not developed in a vacuum. Lobbying by those calling for greater restrictions of the exotic pet trade is typically heavily opposed by those with vested interests, including pet dealers, hobbyists, and a small number of trade-supporting veterinarians [183]. During the interviews, civil servants anticipated or had experienced such opposition to positive lists. Fundamentally, if there are to be restrictions on pet trading and keeping, then the industry tends to favour negative rather than positive lists [184], which in itself may imply recognition by the trade that negative lists are inherently less restrictive than positive lists.

At the national or state/province level, the criteria for species inclusion on negative lists are rarely published although these can be discerned by the types of species prohibited. For example, the rationale for banning a list of non-native species is likely for the purpose of preserving local biodiversity. However, it is without question that at national and state/province levels in both Europe and North America, most restrictions on the trade and ownership of certain species are to protect the public from animal-inflicted injuries. In general, criteria for positive lists are also seldom explicit although, in some cases, these can be discerned from the way legislation is drafted. For instance, where captive-bred animals are permitted, it is likely the list was formulated with species conservation and animal welfare in mind. Likewise, where non-venomous snakes are positively listed, we can infer that public safety was a factor. Indigenous species do not feature on positive lists in European countries given that they are generally well-protected by international law. However, legal protection of indigenous fauna appears less stringent in North America where, for example, in Arkansas and Colorado in the US and in Alberta, Canada, there are positive lists of native species that can be taken from the wild in specified quotas to be kept as pets [185,186].

There is a predictable similarity between the positive lists of Europe and North America as far as mammals are concerned. For example, dogs, cats, rodents, and other small mammals are typically listed. Perhaps the most striking exception to this is in the US state of Nevada where species that appear on a positive list include elephants, primates, and marine mammals [163].

The positive list for reptiles in Flanders, Belgium is notable for its specificity and length. At the time of writing, the list contained 422 reptile species (66 turtle and tortoise sp., 249 lizard sp., and 107 snake sp.) that can be traded or kept. The criteria used to formulate the Flemish list is described on the government’s website as those species that are easy to keep; do not pose a risk to people; that have sufficient husbandry guidance available [187]. However, the established scientific consensus is that reptile husbandry is highly complex [188,189,190,191] and carries significant risks to public health [89,192,193,194], thus the scientific rationale founding the Flemish positive list for reptiles is highly questionable.

The Flemish Government’s website further states that the positive list mainly includes species that are commonly available and frequently kept, and also refers to the involvement of reptile traders and hobbyists in formulating the list [187], thus the issues of impartiality and objectivity are also raised. We speculate that undue influence exerted by reptile traders and hobbyists may have resulted in the preservation of a wide diversity of permitted species, as well as the trade-favourable reference on the government’s website to some reptile species as being “easy to keep”.

Although positive lists, at times, appear to indulge trade interests, they at least ensure that additional or newly described species cannot be traded or kept. Such protections do not exist when whole taxonomic classes of animals are included on positive lists, as is often the case where fishes, amphibians, and reptiles are concerned. Blanket listing of species may also reflect a lack of government expertise in areas that extend beyond birds and mammals—an issue that was broached by civil servants during the interviews.

The protracted Flemish list is in stark contrast to the positive list for reptiles in Norway, which was implemented two years earlier, and which comprises a total of 19 species (2 tortoise sp. and 1 turtle sp., 7 lizard sp., and 9 snake sp. [155]). In formulating the list, the Norwegian Government excluded species that: are poorly adapted to captivity; require specialist care; are typically wild-caught; are capable of transmitting zoonotic diseases to people and other animals; pose a risk to public safety [195]. Given that reptiles are generally reported as being poorly adapted to captivity [188], require specialist care [28], and carry zoonotic disease risks [75,76], then assessment using these criteria would have to involve questions of degree rather than absolutes. Historically, the Norwegian Government has adopted a rigorous and evidence-based approach to regulating selling and keeping of exotic pets. Legislation dating back to 1976 prohibited, on animal welfare grounds, the trading and keeping of exotic pets [196]. Despite the ban, it was reported that importation, trading, and keeping of amphibians and reptiles persisted on a substantial scale, which the Norwegian authorities found unfeasible to eradicate. It was hoped that the positive list for reptiles would reduce illegal trade and that keepers would be more inclined to seek veterinary attention for their animals without fear of prosecution. In legitimising a relatively small part of the reptile trade, the government recognised the potential for negative consequences, but its view was that simply continuing with the ban would not alleviate problems [197]. It remains to be seen whether the positive list will represent an improvement for animal welfare when compared to the previous ban.

As one of the longest established positive lists, the Belgian list for mammals provides a useful case study. Following a claim made by the Belgian Government that the regulation had proven to be effective in a number of important areas [198], an impact assessment was conducted by Eurogroup for Animals in 2016. When compared to data from other European countries, it was found that the positive list had reduced exotic mammal trade overall, and online trade in prohibited species was minimal. During the 2009–2014 period, only 129 exotic mammals belonging to 29 non-listed species had been recorded as confiscated, handed into rescue centres, or found as strays. Public awareness of the positive list gradually increased since its first implementation in 2001. Each confiscation of a non-listed species was widely publicised by the government and it took an estimated ten years for a high level of public familiarity with the positive list to be established [199]. This elevated public awareness correlates not only with greater compliance, but also with ease of enforcement, because members of the public assist the authorities by reporting evidence of contraventions [150,198]. The Belgian mammal list example appears to suggest that reducing species in trade makes law enforcement more feasible.

The Croatian Nature Protection Act regulates the trade in invasive species (animals and plants) via a positive list containing an extensive list of invertebrate and fish species, and 114 bird species. Under the Act, anyone who offers animals for sale is obliged to display a notice to the effect that the animals are potentially invasive and can have multiple negative impacts on biodiversity, the economy, and human health. The Spanish Government has taken a different approach and produced a negative list of potentially invasive species for which a scientifically based risk assessment must be carried out by traders as part of an application to import animals [200]. The EU Regulation (1143/2014) on Invasive Alien Species has been strongly criticised for not being sufficiently proactive or wide-ranging [201,202].

### 4.3. Species Selection for Positive Lists

It is almost inevitable that positive lists for pet trading and keeping will vary between regions, based on a number of factors. The pet keeping culture of an area has an important bearing on which species are listed, because there would be little point in assessing species that were not likely to be sold and kept. It should also be self-evident that species already on negative lists should be excluded from further deliberation. Climate is an important factor if animals are to be kept in spacious outdoor enclosures that benefit their welfare—although their inclusion on positive lists should be further contingent on their invasive potential. The overriding principle is that species included on positive lists should be those that, according to the latest scientific evidence, can be competently kept by an average member of the public in an ordinary domestic setting, and consistent with modern understanding of animal welfare, environmental, and public health and safety considerations. There should be sufficient objective scientific evidence to demonstrate that animals deemed suitable to be kept as pets can have all of their welfare needs met in normal domestic environments. This includes the ability to express a full, normal range of species-typical natural behaviours; provision of a suitable diet and appropriate environmental enrichment; manifestations of abnormal or captivity-stress-related behavioural and negative physical consequences must be absent or rare. It is also important that the practice of keeping those animals is able to be effectively overseen by the relevant authorities, thus species should only be included on positive lists where governments have direct scientific and managemental understanding of those animals.

#### Species Assessment Systems

Several species assessment systems or tools have emerged to assist governments and others in formulating positive lists for pet trading and keeping. During the interviews, civil servants highlighted the lack of departmental expertise to formulate positive lists and, although not specifically mentioned, resource constraints are also likely to be a consideration. Whilst it is not the purpose of this paper to criticise or recommend any particular assessment system, we briefly review each one for reader reference.

The first species assessment tool, published in 2000 by Schuppli and Fraser [203], sets out an ethical framework to guide decision-making and uses a checklist of twelve questions to assess suitability of species as pets. The questions comprehensively cover issues such as the animal’s physical and behavioural characteristics; availability of appropriate husbandry guidance and veterinary services; risks that the animal may pose to public health and the environment; and the animal’s invasive potential and conservation status. Substantial knowledge of the species is required in order to answer the questions, and the relevant issues have to be weighed subjectively, so answers may be biased by personal perspectives. Schuppli, Fraser, and Bacon (2014) build upon the original framework of Schuppli and Fraser (2000) with additional considerations for keeping exotic pets [18]. Ethical concerns relating to the ability of pet animals to function well biologically and to lead fairly natural lives are discussed alongside issues regarding the zoonotic and invasive potential of the animals.

A system for assessing the suitability of mammal species as pets was developed by Wageningen University in 2013 [204]. The method involved gathering information from scientific literature relating to the behavioural ecology, health, welfare, and human–animal relationship of each assessed species and subjecting it to a three-stage assessment process. The first scientific team gathered data for each species in the form of “concise statements” from a limited number of sources and within a restricted timeframe. The second scientific team graded these concise statements—for example, “cannot live outside of a group” was graded according to how critical such a factor would be to its suitability as a pet. The third scientific team reviewed summaries of the above information for each species and determined their suitability as pets. Although discrepancies in assessments between scientists were corrected using statistical methods, this system is largely consensus-based and also costly to execute.

A part algorithm-based system called “EMODE” [205], borrows from the systems of both Schuppli and Fraser and of Koene, and categorises animals according to whether they are “easy”, “moderate”, “difficult” or “extreme” to keep as pets. The EMODE system was designed for both laypersons and scientists. Classes of animals are allocated pre-weighted scores based on issues such as species sensitivity, potential animal longevity, degree of specialised dietary and habitat needs, and risks to human health and safety. The user is required to answer six closed questions for which careful research is necessary. The six answers are combined with pre-weighted scores and the resultant category indicates the level of challenge involved in keeping the animal. EMODE scores do not take into account either the conservation status of a species or its invasive potential. An EMODE website with species already pre-scored is available [191].

Warwick and Steedman [206] propose an objective methodology to assess species suitability for pet keeping, which aims to eliminate or minimise the requirement for consensus-based decision-making. The system requires binary yes/no answers to two sets of five questions, the first of which relates to animal welfare and species conservation and the second set to public health and safety, and risk of invasiveness. System users are required to undertake some basic research in order to answer the questions and affirmative answers are required for all of the questions in order for an animal to be included on a positive list. The system was designed to avoid problems that arise when opinion-based decision-making involving expert committees or those with competing interests produce non-repeatable results. Where information is not readily available, it is recommended that the animal is afforded the benefit of the doubt and not listed.

### 4.4. Comparison of Negative versus Positive Lists

For many species traded as exotic pets, little is known about their biology, natural history, wild population status, invasive potential or risks that they pose to public health. Therefore, it follows that the majority of these species are not included on CITES appendices or restricted via other negative lists due to a lack of research-based information. Most species that are negatively listed present risks to public safety that are clearly discernible and do not require in-depth consideration or analysis.

Historically, far less was known or understood about animal welfare, behaviour, and biology, zoonotic disease risks, and threats to biodiversity, and thus these areas tend not to be accounted for when formulating negative lists. Additionally, a lack of regard for recent scientific research is evident in the fact that most negative lists do not encompass fishes, amphibians, and reptiles, which make up the vast majority of species in trade (except when public safety issues are apparent).

The inadequacy of negative lists can be shown when inverting positive lists. For example, there are estimated to be at least 291 mammal species in the European pet trade [207], but Luxembourg and Belgium have found just 10–14% of these species to be suitable for pet trading and keeping. Applying rigorous scientific assessments to trade across all taxa would likely result in hundreds or thousands of species being added to negative lists. Regardless of this hypothetical scenario, even the most exhaustive negative lists could not accommodate perennial shifts in exotic animal keeping trends, which may present new threats. Therefore, procedural obligations for governments to follow the science means that retaining the negative list model for pet trading and keeping is untenable both in terms of management and cost burdens. Positive lists, on the other hand, can be easily and economically maintained in the light of new evidence.

The sheer scale and diversity of animals currently traded as exotic pets under negative list systems represent a considerable enforcement burden. During the interviews, civil servants reported how their governments struggled to safeguard animal welfare when fundamentally unsuitable species were widely available in trade, and described the challenge involved in proving animal welfare contraventions when so little is known about these species. Specialist training of enforcement personnel at ports is needed, and sometimes manuals and online sources have to be referred to, and outside expertise sought, in order to match species against descriptions on paperwork [208]. Online trade in wild animals as pets and the increased use of social media platforms for this purpose [209,210,211] has developed faster than the capacity of enforcement authorities to track, monitor, and regulate it [212]. Much of the trade, therefore, continues to operate underground [16,213].

Concise positive lists can act as a useful mechanism to control online trade, as has been demonstrated in Belgium where members of the public promptly alert authorities to online advertisements for prohibited species [198]. A positive list also removes “hiding places” for illegal trade—for example, at ports of entry where, currently, representational problems occur when species are misdescribed or paperwork is forged.

A further widespread problem is the complexity of legislation concerning exotic pet trading and keeping, which makes it difficult to understand which species can be legally sold and kept. In order to compile permitted or restricted species lists for this article, it was often the case that several statutes administered by different departments of a government had to be checked. Therefore, it is possible, that such a lack of clarity reduces public compliance. A clear list of permitted species removes the need for enforcement officers to check the legality of species against numerous statutes. Implementing and operating positive lists offers significant cost-savings and is an efficient use of limited resources directed at tackling illegal trade.

Positive lists allow for the development of more effective husbandry practices focused on the select number of species that, according to the latest scientific evidence, thrive in the home environment. Likewise, economies of scale may benefit those that manufacture and sell accessories and equipment in larger quantities to meet the requirements of a small number of pet species. As the exotic pet trade is currently dominated by a relatively small number of species, it is unclear whether, overall, it would be adversely impacted by positive lists.

Negative lists are inherently reactive and require constant reviews and updates in order to keep pace with new species coming into trade. Under negative list systems, problems relating to animal welfare, species conservation and environmental protection, public health and safety, and invasive alien species continue to burgeon [10,27,214,215,216,217,218,219,220,221]. Very often, research into the negative impacts of the exotic pet trade is funded, and limited, by charitable donations and related bodies. Even when evidence is abundant and clearly understood, bureaucratic inertia may entail years of delay in, for example, gaining animal welfare protection or in prohibiting species that are causing ecological harm. Furthermore, any proposed restrictions are likely to be met with vigorous opposition from the pet trade. When species unknown to science are exploited, it leaves open the possibility that species may be traded to extinction before even being described.

Positive lists, on the other hand, do not include species unless rigorous scientific assessment has shown them to be suitable to be traded and kept as pets. This ensures that animals, consumers, and the wider environment are protected. Table 4 summarises the comparison between negative list and positive list systems.

### 4.5. Study Limitations and Future Research

Our analysis of positive lists for pet trading and keeping in Europe and North America was subject to inherent limitations. The concept of positive lists, as a means to regulate pet trading and keeping, is briefly referred to in the scientific literature, usually in relation to species assessment protocols [26,203,204,205]. However, given the relatively recent emergence of positive lists, little has been published, particularly with regard to their application and real-world impacts [150].

Positive lists at the local or municipality level were restricted to those of which our team were aware. There could potentially be other local positive lists in Canada, as well as in the US and Europe, that have not come to our attention. However, given that the present authors are very active on the issue of positive lists, it is unlikely that a cluster of positive lists, as seen in the two Canadian provinces of Ontario and Quebec, would be unnoticed.

The poor response rate to the online survey, particularly in North America, meant that we were unable to gauge the overall level of interest in positive lists. Likewise, we were also unable to gather information on positive lists that had already been implemented and capture feedback from civil servants, including their perceptions of the value of their positive lists and the nature of the public response to them. Qualitative data gathered via telephone interviews with civil servants showed commonality between interviewees on a number of themes. However, it is important to bear in mind that the sample was selected for interview based on their pre-existing interest in positive lists, thus views expressed may not be representative of governments either in Europe or more widely.

To our knowledge, this is the first peer-reviewed evaluation of positive lists as a means to regulate the exotic pet trade. Therefore, these findings may serve as an introductory basis for further research and aid foundational work. As positive lists become more established, there is a clear need for research to assess their impacts in terms of preventing diverse present and future harms. This research would ideally be conducted on the basis of sufficient published data being available to facilitate comparison with previous negative list-based regulation, and between positive lists of different authorities.

### 4.6. Recommendations for Developing and Implementing Positive Lists

The following recommendations draw on the collective experience and observations of the authors in liaising with governments regarding positive lists:

- Species selection criteria should take into account animal welfare; public health and safety; risk of invasiveness; conservation status and provenance. Availability of good quality, impartial husbandry guidance; local enforcement and veterinary expertise; appropriate rescue facilities should also be considered.
- Regardless of the system or process used to select species, positive lists should be developed by independent parties using scientific, evidence-based, and objective sources. Using an impartial species assessment system reduces suggestion of bias.
- In the interests of fairness, inclusivity, and transparency, species selection criteria should be published along with a description of the assessment processes and tools used.
- Where data on a species under assessment are conflicting, inconclusive or absent, the precautionary principle should apply, and the animal should not be listed.
- There should be a provision to add or remove species in the light of new scientific evidence, for which an application process should be in place.
- The burden of proof for adding species to a positive list should rest with the exploiter, using scientific, objective, and impartial evidence.
- Listings should be at species or sub-species level. Scientific names should be used alongside common names.
- Positive lists should be sufficiently concise for ease of enforcement and public compliance. In the interests of clarity, any negative lists should be subsidiary, and for monitoring purposes only.
- Transitional arrangements in the form of “grandfather provisions” should be in place to allow prohibited animals already in private ownership to be kept until they die. Such animals should be subject to a registration system and selling and breeding from them should be prohibited. Where species fail to meet positive list criteria and continue to be kept under a grandfather provision, guidelines could be applied in an endeavour to mitigate problems regarding animal welfare; public health and safety; species invasive risks.
- There should be no restriction on the ability of rescue shelters and sanctuaries to accommodate unwanted, abandoned or seized animals.
- Exemptions could apply for specialists to keep prohibited species. Such prospective keepers should be required to demonstrate that animals are kept as part of a scientifically managed conservation programme, or that they have a standard of expertise, appropriate facilities, and husbandry regimes to meet a high bar threshold.

It is very important that positive lists are based on well-defined criteria that must be met, because arbitrary decision-making can undermine the effectiveness of regulation. The above criteria should be further divided into relevant categories, where needed, to allow for comprehensive assessment. For example, in determining whether a particular species can thrive as a pet, the five freedoms should be taken into account [205]. Species included on a positive list should not require specialist knowledge or skills in order to keep them.

Whereas absolute application of criteria is desirable, in some cases governments may have to gauge which species pose disproportionate risks, for example, when considering zoonotic disease, which are relevant to all animals. Careful research is required with regard to provenance and, whether species are typically wild-caught or captive-bred, because it is advisable not to take claims made by traders at face value.

Enforcement is reportedly easier when listings are at the species or sub-species level rather than at the genus level or above [12]. It follows that listing species rather than taxonomic groups would also be easier for the public to understand. Scientific names should always be used alongside common names because traded species frequently have several common names. Hybrids, such as domestic dogs crossed with wolves, may be prohibited by stipulating that both of the parental species must be on the positive list, and the same principle can apply to crossbreeds.

## 5. Conclusions

Historical and current problems associated with exotic pet trading and keeping, and their future trajectories, warrant a radical but measured shift in the way the relevant issues are managed. In the absence of unified fundamental and widespread regulatory change, the likelihood remains for ongoing animal suffering; species declines; ecosystem disruption; major public health issues. There is already a substantial body of research to demonstrate that the predominantly negative list-based regulations used by most governments are largely ineffective at controlling a raft of issues associated with pet trading and keeping.

In contrast, indications support the effectiveness of positive lists for regulating pet trading and keeping, and we found no evidence to suggest that positive lists worsen current problems. Positive lists and the precautionary principle in general are almost universally embedded into diverse industrial and professional practices, as well as public activities, and in this regard the exotic pet trade is a peculiarly aberrant exception. It is at the very least reasonable to assume that the protection conferred via the safety testing of inanimate products should extend to the live animal “products” produced and promoted by the pet trade. Applying this precautionary principle would likely have far-reaching and widely beneficial impacts for animals, the environment, and people.

Although greater opportunities for research into the effectiveness of positive list-based regulations will arise with their wider use, we believe that there already exists a sufficient evidence base for policy change towards positive list implementation. Progressive governments appear to recognise the potential of positive lists as an elegant solution to the diversity of problems caused by pet trading and keeping. Given that many of these challenges warrant urgent attention, the fact that there is limited research available should not represent an obstacle to adopting a precautionary approach.

## Figures and Tables

**Table 1:** Breakdown of pet population by region.

Country or Region	Description of Animals	Number of Animals (Million)	Reference
European Union	Ornamental fishes Reptiles Birds Small mammals Cats Dogs	300 7.9 37.2 21.2 77.4 68.5	[23]
United States	Ornamental fishes Reptiles Birds Small mammals Cats Dogs	158 9.4 20.3 14 97.2 89.7	[24]
Canada	Ornamental fishes Reptiles Birds Wild mammals Cats Dogs	unknown 0.4 0.4 0.3 8.3 8.2	[7,25]

**Table 2:** Negative and positive list-based regulation as it pertains to pet trading and keeping. International (including EU) and federal (United States and Canada) conventions and legislation.

Region	Legislation	Negative List	Positive List
International	CITES (Convention on International Trade in Endangered Species of Wild Fauna and Flora)	Regulates international trade in species listed in Appendices to prevent unsustainable exploitation. 183 Parties to CITES from all regions of the world.	
	Bern Convention (Convention on the conservation of European wildlife and natural habitats)	Prohibits deliberate capture, keeping, and internal trade of species on Appendix II and regulates exploitation of species on Appendix III. Ratified by 50 countries in Europe and Africa, as well as the EU.	
	Bonn Convention or CMS (Convention on the Conservation of Migratory Species of Wild Animals)	Prohibits the capture of species listed on Appendix I. 131 Parties from all regions of the world.	
European Union	EU Wildlife Trade Regulations (Council Regulation (EC) No. 338/97 and Commission Regulation (EC) No. 865/2006)	Implements CITES in the EU but also adopts stricter domestic measures for some species. Some non-CITES species may also be listed in line with EU internal legislation.	
	Wild Birds Directive (Directive 2009/147/EC on the conservation of wild birds)	Implements the Bern Convention in the EU. Prevents the exploitation (including capture and sale) of most naturally occurring wild birds in the European Territory of the Member States.	
	Habitats Directive (Council Directive 92/43/EEC on the conservation of natural habitats and of wild fauna and flora)	Implements the Bern Convention in the EU. Prohibits the capture, keeping and sale of species listed in Annex IV(a).	
	The IAS Regulation (EU Regulation 1143/2014 on Invasive Alien Species)	The list of species of Union concern contains animal species that are not permitted to be intentionally imported, kept, bred, traded, allowed to reproduce or released into the environment.	
United States	Lacey Act (16 U.S.C. §§ 3371 et seq.)	Prohibits the import and shipment between states of species determined to be injurious or invasive. The law covers all wildlife protected by CITES and more.	
	Captive Wildlife Safety Act	Amends the Lacey Act to specifically prohibit the import, export, transport, sale, receipt, acquisition, or purchase of lions, tigers, leopards, snow leopards, clouded leopards, jaguars, cheetahs, and cougars, and all subspecies and hybrids of these species, across State lines or the U.S. border. The Act provides exemptions for certain individuals and entities.	
	Endangered Species Act of 1973 (16 U.S.C. §§ 1531 et seq.)	Implements CITES within the United States. It prohibits the import and export of endangered species of fish or wildlife, the sale of endangered species in interstate or foreign commerce, the “take” of any such species, and the possession, sale, and transport of any species unlawfully “taken”, without a permit.	
	The Wild Bird Conservation Act (16 U.S.C. §§ 4901-4916)	Limits or prohibits imports of certain exotic bird species. With the exception of excluded species and approved lists, the law prohibits the import of CITES-listed exotic birds. The law allows for the issuances of permits, which are generally not available for private “pet” ownership.	Provisions within the Act allow for a list of exempt species from approved captive-breeding programmes to be imported without a permit.
	Bald and Golden Eagle Protection Act (16 U.S.C. § 668-668c)	Prohibits the take, possession, sale or offer to sell, purchase, barter, transport, and export or import, of any bald or golden eagle. The law allows for the issuances of permits, which are not available for private “pet” ownership.	
United States	Migratory Bird Treaty Act (16 U.S.C. §§ 703-712)	Prohibits the taking, killing, possession, transportation, and importation of migratory bird species native to the U.S. or U.S. territories except as authorised by permit.	
	Animal Health Protection Act (7 U.S.C. §§ 8301-8322)	The Animal Health Protection Act authorises the government to prohibit imports of particular animals to prevent the introduction of “any pest or disease of livestock”. Pursuant to the Act, no person may import leopard, African spurred, or Bell’s hingeback tortoises into the United States, and limited bans have been imposed on other species from certain source countries.	
Canada	Wild Animal and Plant Protection and Regulation of International and Interprovincial Trade Act (S.C. 1992, c 52)	Implements CITES agreements, including the import, export, and interprovincial transportation of CITES-listed species, and related permits.	
	Species at Risk Act (S.C. 2002, c 29)	Regulates ownership and possession of extirpated, endangered and threatened native wildlife species.	
	Migratory Birds Convention Act, 1994 (S.C. 1994, c 22)	Implements the 1916 US–Canada Migratory Birds Convention and protects species of birds covered by the Convention.	
	Canada Wildlife Act (R.S.C., 1985, c. W-9)	The Regulations prohibit, without a permit, the possession of any native animal species.	

**Table 3:** Positive Lists in Europe, the United States, and Canada.

Region	Government	Legislation	Animals Covered (by Class)	Relevant Criteria
Europe	Belgium-Brussels	Art 3bis Dierenbescherming en-welzijnswet http://www.ejustice.just.fgov.be/cgi_loi/change_lg.pl?language=fr&la=F&cn=2009071608&table_name=loi	Mammals	Animal welfare, public health and safety, IAS risk, availability of husbandry guidance
	Belgium-Flanders	Art 3bis Dierenbescherming en-welzijnswet http://www.ejustice.just.fgov.be/cgi_loi/change_lg.pl?language=fr&la=F&cn=2009071608&table_name=loi Art 3bis of The Animal Protection and Welfare Act https://dierenwelzijn.vlaanderen.be/positieve-lijst-reptielen	Mammals Reptiles	Animal welfare, public health and safety, availability of husbandry advice
	Belgium-Wallonia	Art 3bis Dierenbescherming en—welzijnswet http://www.ejustice.just.fgov.be/cgi_loi/change_lg.pl?language=fr&la=F&table_name=loi&cn=2018072406	Mammals	Animal welfare, public health and safety, IAS risk, availability of husbandry guidance
	Croatia	Regulation NN 17/2017-404 of 2017 https://narodne-novine.nn.hr/clanci/sluzbeni/2017_02_17_404.html	Birds, Fishes, Invertebrates	IAS risk
	Luxembourg	Animal Protection Act: Grand Ducal Regulation of 2018 https://deiereschutzgesetz.lu/la-loi/chapitre-1-les-principes-generaux/	Mammals, Birds, Reptiles, Amphibians, Fishes, Invertebrates	Animal welfare, public health and safety, IAS risk, availability of husbandry guidance
	The Netherlands	Animals Act 2011 https://wetten.overheid.nl/BWBR0030250/2020-01-01#Hoofdstuk2	Mammals	Methodology to be agreed
	Norway	Regulation on foreign organisms 2018 Regulation prohibiting the import, trading and keeping of exotic animals 2017 https://lovdata.no/dokument/SF/forskrift/2017-05-11-597	Mammals Reptiles	Animal welfare, human and animal health, IAS risk
	Malta	Protection of animals offered in pet shops (minimum standards) regulations 2014. Restrictions apply only to sale of animals. https://legislation.mt/eli/sl/439.16/eng/pdf	Mammals, Birds, Reptiles, Fishes, Invertebrates	Animal welfare, public safety
United States	Alaska	Alaska Admin. Code tit. 5, § 92.029 http://www.akleg.gov/basis/aac.asp#5.92.028	Mammals, Birds, Reptiles	Animal welfare, public health and safety, conservation, IAS risk
	Arkansas	Ark. Admin. Code § 002.00.1- https://apps.agfc.com/regulations/R1.01/	Mammals, Birds, Reptiles, Amphibians, Fishes	Undetermined
	Colorado	2 Colo. Code Regs. §406-11:1103 https://www.sos.state.co.us/CCR/GenerateRulePdf.do?ruleVersionId=6776&fileName=2%20CCR%20406-11	Mammals, Birds, Reptiles, Amphibians, Fishes	Public health and safety, conservation, IAS risk
United States	Delaware	Delaware Admin. Code tit. 3 903 Exotic Animal Regulations https://regulations.delaware.gov/AdminCode/title3/900/903.shtml#P2_29	Mammals, Reptiles	Animal and human health and safety
	Florida	Fla. Admin. Code r. 68A-6.001–68A-6.018 https://www.flrules.org/gateway/RuleNo.asp?title=CAPTIVE%20WILDLIFE&ID=68A-6.003	Mammals, Birds, Reptiles, Amphibians	Undetermined
	Kentucky	Kentucky Administrative Regulations 301 KY ADC 2:081(native wildlife) https://apps.legislature.ky.gov/Law/KAR/301/002/081.pdf 301 KY ADC 2:082(exotic wildlife) https://apps.legislature.ky.gov/Law/KAR/301/002/082.pdf	Mammals, Birds, Reptiles, Amphibians	Undetermined
	Maine	09-137 Me. Code R. § 7-06 https://www.maine.gov/ifw/docs/unrestrictedspecies.pdf https://legislature.maine.gov/statutes/12/title12sec12152.html https://www.maine.gov/ifw/docs/unrestrictedspecies.pdf	Mammals, Birds, Reptiles, Amphibians, Fishes, Invertebrates	Undetermined
	Maryland	Md. Crim. Law § 18-219 http://mgaleg.maryland.gov/2020RS/Statute_Web/ghg/18-219.pdf Md. Code Regs. 08.03.11.04 http://www.dsd.state.md.us/comar/comarhtml/08/08.03.11.04.htm	Mammals Reptiles, Amphibians	Public health and safety
	Massachusetts	321 Mass. Code Regs. 9.01-9.02 https://www.mass.gov/regulations/321-CMR-900-exemption-list#9-01-exemption-list%20%20https://www.mass.gov/regulations/321-CMR-900-exemption-list#9-02-list-of-domestic-animals	Mammals, Birds, Reptiles, Amphibians, Fishes	Public health and safety, conservation, IAS risk, animal welfare
	Montana	Mont. Code § 87-5-706 https://leg.mt.gov/bills/mca/title_0870/chapter_0050/part_0070/section_0060/0870-0050-0070-0060.html	Mammals, Birds, Reptiles, Amphibians, Fishes, Invertebrates	Public health and safety, IAS risk
	Nevada	Nev. Admin. Code 503.140 https://www.leg.state.nv.us/nac/nac-503.html#NAC503Sec140	Mammals, Birds, Reptiles, Amphibians, Fishes, Invertebrates	Undetermined
	New Hampshire	N.H. Code Admin. R. Fis 804.02 http://www.gencourt.state.nh.us/rules/state_agencies/fis800.html	Mammals, Birds, Reptiles, Amphibians, Fishes	Undetermined
	New Jersey	NJ ADC 7:25-4.4 Exempted species https://advance.lexis.com/documentpage/?pdmfid=1000516&crid=79b79961-6cda-42be-b8b1-57213a98386a&nodeid=AAKACQAAFAAF&nodepath=%2FROOT%2FAAK%2FAAKACQ%2FAAKACQAAF%2FAAKACQAAFAAF&level=4&haschildren=&populated=false&title=%C2%A7+7%3A25-4.4+Exempted+species&config=00JAA1YTg5OGJlYi04MTI4LTRlNjQtYTc4Yi03NTQxN2E5NmE0ZjQKAFBvZENhdGFsb2ftaXPxZTR7bRPtX1Jok9kz&pddocfullpath=%2Fshared%2Fdocument%2Fadministrative-codes%2Furn%3AcontentItem%3A5XKV-PW41-JBDT-B0CP-00008-00&ecomp=c38_kkk&prid=e21ca592-d91e-4da8-8654-357c0ae29d60	Mammals, Birds, Reptiles, Amphibians	Undetermined
United States	North Dakota	N.D. Admin. Code. 48.1-09-01-02 https://www.legis.nd.gov/information/acdata/pdf/48.1-09-01.pdf	Mammals, Birds, Reptiles, Amphibians, Fishes, Invertebrates	Public health and safety, IAS risk
	Oklahoma	Okla. Admin. Code 800:25-25-3 http://www.oar.state.ok.us/oar/codedoc02.nsf/frmMain?OpenFrameSet&Frame=Main&Src=_75tnm2shfcdnm8pb4dthj0chedppmcbq8dtmmak31ctijujrgcln50ob7ckj42tbkdt374obdcli00_	Mammals, Birds, Reptiles, Amphibians, Fishes, Invertebrates	Undetermined
	Rhode Island	250-RICR-40-05-3 3.17 Appendix A: List of Exempt Exotic Animals and Native Wild Animals http://www.dem.ri.gov/pubs/regs/regs/agric/wildanml16.pdf	Mammals, Birds, Reptiles, Fishes, Invertebrates	IAS risk
	Tennessee	Tenn. Comp. R. & Regs. 1660-01-18-.02 Tenn. Code Ann. § 70-4-403 https://www.tn.gov/content/dam/tn/twra/documents/law-enforcement/TennCode_70_4_403.pdf	Mammals, Birds, Reptiles, Amphibians, Fishes	Undetermined
	Utah	Utah Admin. Code r. R657-3-2 https://rules.utah.gov/publicat/code/r657/r657-003.htm#T2	Mammals, Birds	Undetermined
	Vermont	16-4 Vt. Code R. § 116 Wild Bird and Animal Importation and Possession Unrestricted Wild Animal List. https://vtfishandwildlife.com/sites/fishandwildlife/files/documents/Learn%20More/Living%20with%20Wildlife/Importation/Domestic_Species_List.pdf Wild Bird and Animal Importation and Possession Domestic Species List https://vtfishandwildlife.com/sites/fishandwildlife/files/documents/Learn%20More/Living%20with%20Wildlife/Importation/Unrestricted_Wild_Animal_List.pdf	Mammals, Birds, Reptiles, Amphibians, Invertebrates	Public health and safety, IAS risk, suitability as pets
	Wisconsin	Wis. Stat. Ann. §169.04 https://docs.legis.wisconsin.gov/statutes/statutes/169/04/4/a	Reptiles, Amphibians, Mammals, Birds, Invertebrates	Undetermined
	Wyoming	Wyo. Admin. Code 040.0001.10 § 3 https://rules.wyo.gov/Search.aspx?mode=1 (Search Game and Fish Commission, Chapter 10)	Mammals, Birds, Reptiles, Amphibians, Fishes, Invertebrates	Undetermined
Canada (Provinces and Territory)	Alberta	Wildlife Act RSA 2000, c W-10; Wildlife Regulation (Alta Reg 143/1997) https://www.canlii.org/en/ab/laws/regu/alta-reg-143-1997/latest/alta-reg-143-1997.html	Mammals, Birds, Amphibians	Undetermined
	New Brunswick	Exotic Wildlife Regulation—Fish and Wildlife Act https://www.canlii.org/en/nb/laws/regu/nb-reg-92-74/latest/nb-reg-92-74.html#document	Mammals, Birds, Reptiles, Amphibians	Animal welfare, species conservation, IAS risk, public safety
	Newfoundland and Labrador	Wild Life Act, RSNL 1990, c W-8 https://www.canlii.org/en/nl/laws/regu/cnlr-1156-96/latest/cnlr-1156-96.html#document	Mammals, Birds, Reptiles, Amphibians, Fishes, Invertebrates	IAS risk
Canada (Provinces and Territory)	Nova Scotia	Section 113(at) of the Wildlife Act, R.S.N.S. 1989, c504 and Section 6 of the General Wildlife Regulations—Captive Wildlife Permit And Import Permit Exclusion List https://novascotia.ca/natr/wildlife/laws/pdf/App2.pdf	Mammals, Birds, Reptiles, Amphibians	Public health, species conservation
	Saskatchewan	The Captive Wildlife Regulations, RRS c W-13.1 Reg 13 https://www.canlii.org/en/sk/laws/regu/rrs-c-w-13.1-reg-13/latest/rrs-c-w-13.1-reg-13.html	Mammals, Birds, Reptiles, Amphibians, Fishes	Undetermined
	Nunavut	Wildlife Act 2003 Wildlife Act 1988 (replaced) Wildlife Genera Regulations 1992 https://www.canlii.org/en/nu/laws/stat/snu-2003-c-26/latest/snu-2003-c-26.html	Reptiles, birds	Undetermined
Canada (Towns and municipalities)	City of Kitchener	Animals—Regulation Chapter 408, 2016 https://lf.kitchener.ca/WebLinkExt/0/doc/1497603/Page1.aspx	Mammals, Birds, Reptiles, Amphibians, Fishes, Invertebrates	Undetermined
	Town of Aurora	Town of Aurora, By-law Number 61 97-1 9, 2019 https://www.aurora.ca/en/your-government/resources/by-laws/6197-19-Animal-Services-By-law.pdf	Mammals, Birds, Reptiles, Amphibians, Fishes, Invertebrates	Undetermined
	Town of Newmarket	Town of Newmarket, By-law 2020-30 https://www.newmarket.ca/LivingHere/Documents/2020-30%20Animal%20Control%20By-law.pdf	Mammals, Birds, Reptiles, Amphibians, Fishes, Invertebrates	Public health and safety
	Brossard	Règlement 219 relatif au controle des animaux https://www.brossard.ca/in/rest/annotationSVC/Attachment/attach_cmsUpload_65f94abb-63d1-419c-8d01-e5ca7bc759b1	Mammals, Birds, Reptiles, Amphibians, Fishes	Undetermined
	Chateauguay	Règlement G-018-17 relatif aux animaux et abrogeant le chapitre XIV du règlement G-2000 https://www.ville.chateauguay.qc.ca/sites/default/files/G_018-17_animaux_dangereux.pdf	Reptiles, Amphibians, Invertebrates	Public safety
	Chicoutimi/ Saguenay	Règlement VS-R-2007-50 concernant les animaux sur le territoire de la ville de Saguenay https://ville.saguenay.ca/files/reglements_municipaux/animaux/ca_vs_r_2007_50.pdf	Mammals, Birds, Reptiles, Amphibians, Fishes	Undetermined
	Gatineau	Règlement numéro 183-2005 concernant la garde, le contrôle et le soin des animaux dans les limites de la ville de Gatineau http://www.tantelori.com/PDFs/PetLicense-FR-Gatineau-Regs-R-0183-2005-to-2012-04-23%20-Animaux%20(french-francais).pdf C-61.1, r. 5—Regulation respecting animals in captivity	Mammals, Birds, Reptiles, Amphibians, Fishes, Invertebrates	Public safety
	Laval	Règlement numéro l-12430 Concernant les animaux 2017 https://www.laval.ca/Documents/Pages/Fr/Citoyens/reglements/reglements-codifies/reglement-l-12430.pdf	Mammals, Birds, Reptiles, Amphibians	Public safety
Canada (Towns and municipalities)	Longueil	Règlement co-2008-523 sur le Contrôle des Animaux 2008 https://www.longueuil.quebec/sites/longueuil/files/reglements/co-2008-523_original.pdf	Mammals, Birds, Reptiles, Fishes	Undetermined
	Montréal	Règlement sur le Contrôle des Animaux 16-060 2016 http://ville.montreal.qc.ca/sel/sypre-consultation/afficherpdf?idDoc=27628&typeDoc=1	Mammals, Birds, Reptiles, Amphibians	Public safety, species conservation, animal welfare
	Québec	Règlement sur les animaux domestiques (R.V.Q 1059) https://reglements.ville.quebec.qc.ca/fr/showdoc/cr/R.V.Q.1059	Mammals, Birds, Reptiles, Fishes	Public safety
	Rimouski	Règlement 44-2002 concernant les animaux https://rimouski.ca/storage/app/media/ville/administration/reglements-municipaux/Reglement_1094-2018.pdf	Mammals, Birds, Reptiles, Fishes	Undetermined
	Saint-Hyacinthe	Règlement numéro 30 relatif aux animaux https://www.ville.st-hyacinthe.qc.ca/medias/services-aux-citoyens/reglementations/Regl30.pdf	Mammals, Birds, Reptiles, Fishes	Undetermined
	Saint-Jean-Sur-Richelieu	Règlement no. 0771 concernant la garde des animaux et abrogeant les règlements nos. 0291 et 0441 https://sjsr.ca/wp-content/uploads/2019/06/codification-administrative-1742.pdf	Mammals, Birds, Reptiles, Fishes	Public safety
	Shawinigan	Règlement municipal, Titre 8: Garde et controle des animaux http://www.shawinigan.ca/Document/Fichiers%20PDF/Ville/Reglements/SH-1/Titre%208%20animaux%20(190107).pdf	Mammals, Birds, Reptiles, Amphibians, Fishes, Invertebrates	Undetermined
	Sherbrooke	Règlement no. 1, Titre 5, chap. 10, sec. 2 https://contenu.maruche.ca/Fichiers/3337a882-4a53-e611-80ea-00155d09650f/Sites/333dd3d3-915d-e611-80ea-00155d09650f/Documents/Reglements%20municipaux/reglement-1.pdf	Mammals, Birds, Reptiles, Amphibians, Fishes	Public safety
	Trois-Rivières	Règlement sur la garde d’animaux (2014, chapitre 158) https://contenu.maruche.ca/Fichiers/d477a882-4a53-e611-80ea-00155d09650f/Sites/742ceda8-915d-e611-80ea-00155d09650f/Documents/Règlements/Reglement_sur_la_garde_d_animaux.pdf	Mammals, Birds, Reptiles, Fishes	Public safety

**Table 4:** Comparison of negative list and positive list systems for pet trading and keeping.

Negative List System	Positive List System
No evidence base to show that permitted species are suitable pets, in terms of animal health and welfare, and safety of people and the environment.	Evidence-based species risk assessments offer consumer protection, animal health and welfare, and environmental safeguards.
Administrative complexity and difficulty for enforcers. Array of legislation and enforcement protocols requiring a high level of expertise.	Administrative simplicity and ease for enforcers. Greater clarity for public regarding which species can be legally kept assists compliance.
Wide diversity of permitted species with largely unreliable husbandry guidance or species that may be unable to thrive in captivity.	Reliable, and improving, husbandry guidance for established pet species.
Authorities forced to be reactive as new species come into trade or problems are identified. Burden of proof is on individual scientists, humane and other groups, societies, or governments.	Authorities able to take proactive measures. Species added to positive lists only after thorough risk assessments have been undertaken. Burden of proof for adding species is on the prospective exploiter.

## Supplementary Materials

The following are available online at https://www.mdpi.com/2076-2615/10/12/2371/s1, Supplementary File: Questionnaire A, Questionnaire B, Telephone Interview Questions.

## References

[1] Borzée A., McNeely J., Magellan K., Miller J.R., Porter L., Dutta T., Kadinjappalli K.P., Sharma S., Shahabuddin G., Aprilinayati F. (2020). COVID-19 highlights the need for more effective wildlife trade legislation. Trends Ecol. Evol..

[2] Gibbons K. (2020). End Exotic Pet Trade to Stop Diseases Spreading, Stars Tell Boris Johnson. The Times.

[3] Hance J. (2018). Will Trade Bans Stop a Deadly Salamander Plague from Invading the US?. Mongabay.

[4] Brown R. (2006). Exotic pets invade United States ecosystems: Legislative failure and a proposed solution. Ind. LJ.

[5] Soulsbury C.D., Iossa G., Kennell S., Harris S. (2009). The welfare and suitability of primates kept as pets. J. Appl. Anim. Welf. Sci..

[6] Eurogroup for Animals (2020). Analysis of National Legislation Related to the Keeping and Sale of Exotic Pets in Europe. https://www.eurogroupforanimals.org/sites/eurogroup/files/2020-07/Eurogroup%20for%20Animals_Exotic%20pets%20reoprt_v5.pdf.

[7] World Animal Protection Risky Business: The Unregulated Exotic Pet Trade in Canada. https://www.worldanimalprotection.ca/sites/default/files/media/ca_-_en_files/wap_exotic_pets_in_canada_report_final_forweb_oct_3_2019.pdf.

[8] Smith K., Zambrana-Torrelio C., White A., Asmussen M., Machalaba C., Kennedy S., Lopez K., Wolf T., Daszak P., Travis D. (2017). Summarizing US wildlife trade with an eye toward assessing the risk of infectious disease introduction. EcoHealth.

[9] Green J., Coulthard E., Norrey J., Megson D., D’Cruze N. (2020). Risky business: Live non-CITES wildlife UK imports and the potential for infectious diseases. Animals.

[10] Auliya M., Altherr S., Ariano-Sanchez D., Baard E.H., Brown C., Brown R.M., Cantu J.-C., Gentile G., Gildenhuys P., Henningheim E. (2016). Trade in live reptiles, its impact on wild populations, and the role of the European market. Biol. Conserv..

[11] Biondo M.V., Burki R.P. (2020). A systematic review of the ornamental fish trade with emphasis on coral reef fishes—an impossible task. Animals.

[12] Marshall B.M., Strine C., Hughes A.C. (2020). Thousands of reptile species threatened by under-regulated global trade. Nat. Commun..

[13] Biondo M.V. (2017). Quantifying the trade in marine ornamental fishes into Switzerland and an estimation of imports from the European Union. Glob. Ecol. Conserv..

[14] UNEP, WCMC (2008). Monitoring of International Trade in Ornamental Fish.

[15] CITES Trade Database. https://trade.cites.org.

[16] Karesh W.B., Cook R.A., Gilbert M., Newcomb J. (2007). Implications of wildlife trade on the movement of avian influenza and other infectious diseases. J. Wildl. Dis..

[17] Norconk M.A., Atsalis S., Tully G., Santillán A.M., Waters S., Knott C.D., Ross S.R., Shanee S., Stiles D. (2020). Reducing the primate pet trade: Actions for primatologists. Am. J. Primatol..

[18] Schuppli C.A., Fraser D., Bacon H. (2014). Welfare of non-traditional pets. Rev. Sci. Tech..

[19] Carpenter A.I., Andreone F., Moore R.D., Griffiths R.A. (2014). A review of the international trade in amphibians: The types, levels and dynamics of trade in CITES-listed species. Oryx.

[20] AVMA Pet Ownership Is on the Rise. https://www.avma.org/blog/pet-ownership-rise.

[21] Lockwood J., Welbourne D., Romagosa C., Cassey P., Mandrak N., Strecker A., Leung B., Stringham O., Udell B., Episcopio-Sturgeon D. (2019). When pets become pests: The role of the exotic pet trade in producing invasive vertebrate animals. Front. Ecol. Environ..

[22] Pet Food Manufacturers’ Association UK Pet Population Statistics. https://www.pfma.org.uk.

[23] Fediaf European Facts & Figures 2019. http://www.fediaf.org/images/FEDIAF_facts_and_figs_2019_cor-35-48.pdf.

[24] APPA Pet Industry Market Size & Ownership Statistics. https://www.americanpetproducts.org/.

[25] Bedford E. Number of Cats and Dogs in Households in Canada in 2018. https://www.statista.com/statistics/1015882/number-of-pet-cats-and-dogs-canada/.

[26] Warwick C., Steedman C., Jessop M., Arena P., Pilny A., Nicholas E. (2018). Exotic pet suitability: Understanding some problems and using a labeling system to aid animal welfare, environment, and consumer protection. J. Vet. Behav..

[27] Scheffers B.R., Oliveira B.F., Lamb I., Edwards D.P. (2019). Global wildlife trade across the tree of life. Science.

[28] Whitehead M.L. (2018). Factors contributing to poor welfare of pet reptiles. Testudo.

[29] Brook B.W., Sodhi N.S. (2006). Rarity bites. Nature.

[30] Stuart B.L., Rhodin A.G.J., Grismer L.L., Hansel T. (2006). Scientific description can imperil species. Science.

[31] Menegon M., Davenport T.R., Howell K.M. (2011). Description of a new and critically endangered species of *Atheris* (*Serpentes: Viperidae*) from the Southern Highlands of Tanzania, with an overview of the country’s tree viper fauna. Zootaxa.

[32] Altherr S., Lameter K. (2020). The rush for the rare: Reptiles and amphibians in the European pet trade. Animals.

[33] Böhme W., Ziegler T. (1997). *Varanus melinus* sp. n., ein neuer Waran aus der V. indicus-Gruppe von den Molukken, Indonesien. Herpetofauna.

[34] Altherr S., Lameter K., Carlos Cantu J. (2019). The trade in nationally protected lizards from Australia, Cuba, and Mexico And the EU’s role as a main destination. TRAFFIC Bull..

[35] Ng P.K., Schubart C.D., Lukhaup C. (2015). New species of “vampire crabs” (Geosesarma De Man, 1892) from central Java, Indonesia, and the identity of Sesarma (Geosesarma) nodulifera De Man, 1892 (Crustacea, Brachyura, Thoracotremata, Sesarmidae). Raffles Bull. Zool..

[36] Koehler G., Vesely M. (2011). A new species of Thecadactylus from Sint Maarten, Lesser Antilles (Reptilia, Squamata, Gekkonidae). ZooKeys.

[37] Böhme W., Jacobs H. (2001). *Varanus macraei* sp. n., eine neue Waranart der *V. prasinus*-Gruppe aus West Irian, Indonesien. Herpetofauna.

[38] Altherr S., Freyer D., Lameter K. (2020). Strategien zur Reduktion der Nachfrage nach als Heimtiere gehaltenen Reptilien, Amphibien und kleinen Säugetieren. Artenschutzrelevanz des Heimtierhandels.

[39] Herrel A., van der Meijden A. (2014). An analysis of the live reptile and amphibian trade in the USA compared to the global trade in endangered species. Herpetol. J..

[40] Evers H., Pinnegar J.K., Taylor M.I. (2019). Where are they all from?—Sources and sustainability in the ornamental freshwater fish trade. J. Fish Biol..

[41] Reptiles Magazine Ball Python Morph History. https://www.reptilesmagazine.com/ball-python-morph-history/.

[42] Hedgehog Care 101 How Much Do Hedgehogs Cost?. http://www.hedgehogcare101.com/how-much-do-hedgehogs-cost/.

[43] EcoHealth Alliance Ecohealth Alliance Calls for Improved EDUCATION Surrounding Exotic Pet Ownership. https://www.ecohealthalliance.org/2011/03/ecohealth-alliance-calls-for-improved-education-surrounding-exotic-pet-ownership.

[44] VetEffec T. (2015). Study on the Welfare of Dogs and Cats Involved in Commercial Practices.

[45] OneKind Pet Origins (2014). Giving Our Companions a Better Start in Life: The Case for Reform of UK Pet Vending Legislation.

[46] Bennett P., Howell T. (2013). Pet-Care Practices of Victorian Dog, Cat, Rabbit and Bird Owners: What Issues Should We Be Targeting with Educational Materials? Australian Institute of Animal Management Proceedings. https://52.63.179.249/display/publication82433.

[47] Grant R.A., Montrose V.T., Wills A.P. (2017). ExNOTic: Should we be keeping exotic pets?. Anim. Basel.

[48] Warwick C. (2014). The morality of the reptile” pet” trade. J. Anim. Ethics.

[49] Amaral-Zettler L.A., Schmidt V., Smith K.F. (2018). Microbial community and potential pathogen shifts along an ornamental fish supply chain. Microorganisms.

[50] Laidlaw R. (2005). Scales and Tails: The Welfare and Trade of Reptiles Kept as Pets in Canada; Zoo Check Canada. https://www.zoocheck.com/wp-content/uploads/2016/06/Reptile_Report_FA.pdf.

[51] Endcap Wild Pets in the European Union. https://endcap.eu/wp-content/uploads/2013/02/Report-Wild-Pets-in-the-European-Union.pdf.

[52] Baker S.E., Cain R., Van Kesteren F., Zommers Z.A., D’cruze N., Macdonald D.W. (2013). Rough trade: Animal welfare in the global wildlife trade. BioScience.

[53] Ashley S., Brown S., Ledford J., Martin J., Nash A.E., Terry A., Tristan T., Warwick C. (2014). Morbidity and mortality of invertebrates, amphibians, reptiles, and mammals at a major exotic companion animal wholesaler. J. Appl. Anim. Welf. Sci..

[54] Peta2 Why the International Exotic-Pet Trade Is Actually Worse Than You Thought. https://www.peta2.com/news/international-exotic-pet-trade-germany/.

[55] Peta UK The Heartbreaking Story of How Animals Are Bred for the European Pet Trade. https://www.peta.org.uk/blog/animals-breeders-european-pet-trade/.

[56] Schmid R., Doherr M.G., Steiger A. (2006). The influence of the breeding method on the behaviour of adult African grey parrots (*Psittacus erithacus*). Appl. Anim. Behav. Sci..

[57] Warwick C. (2015). Captive breeding—Saving wildlife? Or saving the pet trade?. Ecol. J..

[58] D’Cruze N., Paterson S., Green J., Megson D., Warwick C., Coulthard E., Norrey J., Auliya M., Carder G. (2020). Dropping the ball? The welfare of ball pythons traded in the EU and North America. Animals.

[59] Engebretson M. (2006). The welfare and suitability of parrots as companion animals: A review. Anim. Welf..

[60] Luescher A.U. (2006). Manual of Parrot Behavior.

[61] Rose M.P., Williams D.L. (2014). Neurological dysfunction in a ball python (*Python regius*) colour morph and implications for welfare. J. Exot. Pet Med..

[62] Arena P.C., Steedman C., Warwick C. (2012). Amphibian and reptile pet markets in the EU: An investigation and assessment. Animal Protection Agency, Animal Public, International Animal Rescue, Eurogroup for Wildlife and Laboratory Animals.

[63] McBride E.A. (2017). Small prey species’ behaviour and welfare: Implications for veterinary professionals. J. Small Anim. Pract..

[64] Costa P., Macchi E., Valle E., Marco M.D., Nucera D.M., Gasco L., Schiavone A. (2016). An association between feather damaging behavior and corticosterone metabolite excretion in captive African grey parrots (*Psittacus erithacus*). PeerJ.

[65] Loeb J. (2018). Reptile illness is caused by bad husbandry. Vet. Rec..

[66] Pees M., Müller K., Mathes K., Korbel R., Seybold J., Lierz M., Krautwald-Junghanns M.-E. (2014). Evaluierung der Haltungsbedingungen häufig gehaltener Reptilienspezies in Deutschland. Kleintierpraxis.

[67] Warwick C., Arena P., Steedman C. (2019). Spatial considerations for captive snakes. J. Vet. Behav..

[68] Arena P.C., Crawford M., Forbes N.A., Frye F.L., Grant R., Howell T., Jessop M., Lambiris A.J.L., Mancera K., Morton D. (2018). The need for snakes to fully stretch. Vet. Rec..

[69] Toland E., Warwick C., Arena P. (2012). Pet hate. Biologist.

[70] Wood E. (2011). Collection of Coral Reef Fish for Aquaria: Global Trade, Conservation Issues and Management Strategies.

[71] Howell T.J., Warwick C., Bennett P.C. (2020). Self-reported snake management practices among owners in Victoria, Australia. Vet. Rec..

[72] Howell T.J., Bennett P.C. (2017). Despite their best efforts, pet lizard owners in Victoria, Australia, are not fully compliant with lizard care guidelines and may not meet all lizard welfare needs. J. Vet. Behav..

[73] Kolby J. To Prevent the Next Pandemic, It’s the Legal Wildlife Trade We Should Worry About. https://www.nationalgeographic.com/animals/2020/05/to-prevent-next-pandemic-focus-on-legal-wildlife-trade/.

[74] Aguirre A.A., Catherina R., Frye H., Shelley L. (2020). Illicit wildlife trade, wet markets, and COVID-19: Preventing future pandemics. World Med. Health Policy.

[75] Chomel B.B., Belotto A., Meslin F.X. (2007). Wildlife, exotic pets, and emerging zoonoses. Emerg. Infect. Dis..

[76] Stull J.W., Brophy J., Weese J. (2015). Reducing the risk of pet-associated zoonotic infections. CMAJ.

[77] Paul M., King L., Carlin E.P. (2010). Zoonoses of people and their pets: A US perspective on significant pet-associated parasitic diseases. Trends Parasitol..

[78] Pilny A., Reavill D. (2020). Emerging and re-emerging diseases of selected avian species. Veterinary Clin. N. Am. Exot. Anim. Pract..

[79] Sklenovská N., Van Ranst M. (2018). Emergence of monkeypox as the most important orthopoxvirus infection in humans. Front. Public Health.

[80] Smith K.F., Behrens M., Schloegel L.M., Marano N., Burgiel S., Daszak P. (2009). Reducing the risks of the wildlife trade. Science.

[81] Colomb-Cotinat M., Le Hello S., Rosières X., Lailler R., Weill F.-X., Jourdan-da Silva N. (2014). Salmonelloses Chez Des Ieunes Enfants et Exposition Aux Reptiles Domestiques: Investigation en France Métropolitaine en 2012.

[82] Hoffmann B., Tappe D., Höper D., Herden C., Boldt A., Mawrin C., Niederstraßer O., Müller T., Jenckel M., van der Grinten E. (2015). A variegated squirrel bornavirus associated with fatal human encephalitis. N. Engl. J. Med..

[83] Arnold K.E., Williams N.J., Bennett M. (2016). ‘Disperse abroad in the land’: The role of wildlife in the dissemination of antimicrobial resistance. Biol. Lett..

[84] Broens E.M., van Geijlswijk I.M. (2018). Prudent use of antimicrobials in exotic animal medicine. Veterinary Clin. N. Am. Exot. Anim. Pract..

[85] Rose S., Hill R., Bermudez L.E., Miller-Morgan T. (2013). Imported ornamental fish are colonized with antibiotic-resistant bacteria. J. Fish Dis..

[86] Lloyd D.H. (2007). Reservoirs of antimicrobial resistance in pet animals. Clin. Infect. Dis..

[87] Reaser J.K., Clark E.E., Meyers N.M. (2008). All creatures great and minute: A public policy primer for companion animal zoonoses. Zoonoses Public Health.

[88] Can Ö.E., D’Cruze N., Macdonald D.W. (2019). Dealing in deadly pathogens: Taking stock of the legal trade in live wildlife and potential risks to human health. Glob. Ecol. Conserv..

[89] Warwick C., Arena P., Steedman C., Jessop M. (2012). A review of captive exotic animal-linked zoonoses. J. Environ. Health Res..

[90] Warwick C., Corning S. (2013). Managing patients for zoonotic disease in hospitals. JRSM Short Rep..

[91] Westgarth C., Brooke M., Christley R.M. (2018). How many people have been bitten by dogs? A cross-sectional survey of prevalence, incidence and factors associated with dog bites in a UK community. J. Epidemiol. Community Health.

[92] Warwick C., Steedman C. (2012). Injuries, envenomations and stings from exotic pets. J. R. Soc. Med..

[93] Lakestani N., Donaldson M.L. (2015). Dog bite prevention: Effect of a short educational intervention for preschool children. PLoS ONE.

[94] Chapman S., Cornwall J., Righetti J., Sung L. (2000). Preventing dog bites in children: Randomised controlled trial of an educational intervention. Bmj.

[95] Smith K.M., Smith K.F., D’Auria J.P. (2012). Exotic pets: Health and safety issues for children and parents. J. Pediatr. Health Care.

[96] BFF USA Search Exotic Incidents Database. https://www.bornfreeusa.org/exotic-incidents-database/.

[97] Schaper A., Desel H., Ebbecke M., De Haro L., Deters M., Hentschel H., Hermanns-Clausen M., Langer C. (2009). Bites and stings by exotic pets in Europe: An 11 year analysis of 404 cases from northeastern Germany and southeastern France. Clin. Toxicol. Phila. Pa.

[98] Schaper A., de Haro L., Ebbecke M., Desel H., Langer C. (2004). Klapperschlangenbisse Vergiftungen durch exotische Haustiere nehmen zu. Dtsch. Arzteblatt-Koln-.

[99] Warrick B.J., Boyer L.V., Seifert S.A. (2014). Non-native (exotic) snake envenomations in the US, 2005–2011. Toxins.

[100] Bush E.R., Baker S.E., Macdonald D.W. (2014). Global trade in exotic pets 2006–2012. Conserv. Biol..

[101] Courchamp F., Angulo E., Rivalan P., Hall R.J., Signoret L., Bull L., Meinard Y. (2006). Rarity value and species extinction: The anthropogenic Allee effect. PLoS Biol..

[102] Holden M.H., McDonald-Madden E. (2017). High prices for rare species can drive large populations extinct: The anthropogenic allee effect revisited. J. Theor. Biol..

[103] Altherr S., Brückner J., Mackensen H. (2010). Mangelhafte Umsetzung der BMELV-Tierbörsen-Leitlinien—Eine Bestandsaufnahme.

[104] Shepherd C.R., Janssen J., Noseworthy J. (2019). A case for listing the Union Island Gecko *Gonatodes daudini* in the Appendices of CITES. Glob. Ecol. Conserv..

[105] Nowak K. The World Has a Chance to Make the Wild Animal Trade More Humane. https://www.nationalgeographic.com/news/2016/02/160226-animal-trade-animal-welfare-exotic-pets-cites-wildlife-trafficking/.

[106] Altherr S. (2014). Stolen Wildlife—Why the EU needs to Tackle Smuggling of Nationally Protected Species.

[107] Janssen J., de Silva A. (2019). The presence of protected reptiles from Sri Lanka in international commercial trade. TRAFFIC Bull..

[108] Ngo H.N., Nguyen T.Q., Van Nguyen T., Van Schingen M., Ziegler T. (2018). Microhabitat selection and communal nesting in the insular Psychedelic Rock Gecko, *Cnemaspis psychedelica*, in southern Vietnam with updated information on trade. Nat. Conserv..

[109] Flecks M., Weinsheimer F., Böhme W., Chenga J., Lötters S., Rödder D. (2012). Watching extinction happen: The dramatic population decline of the critically endangered Tanzanian Turquoise Dwarf Gecko, *Lygodactylus williamsi*. Salamandra.

[110] Nijman V. (2014). Keeping an Ear to the Ground: Monitoring the Trade in Earless Monitor Lizards: A Rapid Assessment.

[111] Janssen J., Leupen B.T. (2019). Traded under the radar: Poor documentation of trade in nationally-protected non-CITES species can cause fraudulent trade to go undetected. Biodivers. Conserv..

[112] United Nations Office on Drugs and Crime (2016). World Wildlife Crime Report: Trafficking in Protected Species.

[113] Sollund R., Maher J. (2015). The Illegal Wildlife Trade: A Case Study Report on the Illegal Wildlife Trade in the United Kingdom, Norway, Colombia and Brazil. A Study Compiled as Part of the EFFACE Project. https://efface.eu/sites/default/files/EFFACE_Illegal%20Wildlife%20Trade_revised.pdf.

[114] Lyons J.A., Natusch D.J. (2011). Wildlife laundering through breeding farms: Illegal harvest, population declines and a means of regulating the trade of green pythons (*Morelia viridis*) from Indonesia. Biol. Conserv..

[115] TRAFFIC Captive Bred or Wild Taken?. https://www.traffic.org/site/assets/files/7446/captive-bred-or-wild-taken.pdf.

[116] Fisher S., Fisher R., Alcaraz S., Gallo-Barneto R., Patino-Martinez C., Jurado L.L., Rochester C. (2019). Life-history comparisons between the native range and an invasive island population of a colubrid snake. Isl. Invasives Scaling Meet Chall..

[117] Mooney H.A., Cleland E.E. (2001). The evolutionary impact of invasive species. Proc. Natl. Acad. Sci. USA.

[118] Chinchio E., Crotta M., Romeo C., Drewe J.A., Guitian J., Ferrari N. (2020). Invasive alien species and disease risk: An open challenge in public and animal health. PLOS Pathog..

[119] Willson J.D., Dorcas M.E., Snow R.W. (2011). Identifying plausible scenarios for the establishment of invasive Burmese pythons (*Python molurus*) in Southern Florida. Biol. Invasions.

[120] Krysko K.L., Burgess J.P., Rochford M.R., Gillette C.R., Cueva D., Enge K.M., Somma L.A., Stabile J.L., Smith D.C., Wasilewski J.A. (2011). Verified non-indigenous amphibians and reptiles in Florida from 1863 through 2010: Outlining the invasion process and identifying invasion pathways and stages. Zootaxa.

[121] Stringham O.C., Lockwood J.L. (2018). Pet problems: Biological and economic factors that influence the release of alien reptiles and amphibians by pet owners. J. Appl. Ecol..

[122] Episcopio-Sturgeon D.J., Pienaar E.F. (2020). Investigating support for management of the pet trade invasion risk. J. Wildl. Manag..

[123] Engeman R., Jacobson E., Avery M.L., Meshaka W.E. (2011). The aggressive invasion of exotic reptiles in Florida with a focus on prominent species: A review. Curr. Zool..

[124] Krysko K.L., Somma L.A., Smith D.C., Gillette C.R., Cueva D., Wasilewski J.A., Enge K.M., Johnson S.A., Campbell T.S., Edwards J.R. (2016). New verified nonindigenous amphibians and reptiles in Florida through 2015, with a summary of over 152 years of introductions. IRCF Reptil. Amphib..

[125] Davenport K., Collins J. (2016). European Code of Conduct on Pets and Invasive Alien Species.

[126] Bergman D., Breck S.W., Bender S. (2009). Dogs Gone Wild: Feral Dog Damage in the United States.

[127] Trouwborst A., McCormack P.C., Martínez Camacho E. (2020). Domestic cats and their impacts on biodiversity: A blind spot in the application of nature conservation law. People Nat..

[128] Cabrera-Pérez M.Á., Gallo-Barneto R., Esteve I., Patiño-Martínez C., López-Jurado L.F. (2012). The management and control of the California kingsnake in Gran Canaria (Canary Islands): Project LIFE+ Lampropeltis. Aliens Invasive Species Bull..

[129] Hulme P.E., Bacher S., Kenis M., Klotz S., Kühn I., Minchin D., Nentwig W., Olenin S., Panov V., Pergl J. (2008). Grasping at the routes of biological invasions: A framework for integrating pathways into policy. J. Appl. Ecol..

[130] The Institute for European Environmental Policy Biodiversity and Ecosystem Services. Invasive Alien Species. https://ieep.eu/work-areas/biodiversity/invasive-alien-species.

[131] Daszak P., Cunningham A.A., Hyatt A.D. (2000). Emerging infectious diseases of wildlife-- threats to biodiversity and human health. Science.

[132] Cunningham A.A., Daszak P., Wood J.L.N. (2017). One Health, emerging infectious diseases and wildlife: Two decades of progress?. Philos. Trans. R. Soc. B Biol. Sci..

[133] Fogell D.J., Martin R.O., Bunbury N., Lawson B., Sells J., McKeand A.M., Tatayah V., Trung C.T., Groombridge J.J. (2018). Trade and conservation implications of new beak and feather disease virus detection in native and introduced parrots. Conserv. Biol. J. Soc. Conserv. Biol..

[134] Varsani A., Regnard G.L., Bragg R., Hitzeroth I.I., Rybicki E.P. (2011). Global genetic diversity and geographical and host-species distribution of beak and feather disease virus isolates. J. Gen. Virol..

[135] Derraik J., Phillips S. (2010). Online trade poses a threat to biosecurity in New Zealand. Biol. Invasions.

[136] Ribeiro L.P., Carvalho T., Becker C.G., Jenkinson T.S., da Leite D.S., James T.Y., Greenspan S.E., Toledo L.F. (2019). Bullfrog farms release virulent zoospores of the frog-killing fungus into the natural environment. Sci. Rep..

[137] Thomas V., Wang Y., Van Rooij P., Verbrugghe E., Baláž V., Bosch J., Cunningham A.A., Fisher M.C., Garner T.W., Gilbert M.J. (2019). Mitigating *Batrachochytrium salamandrivorans* in Europe. Amphib. Reptil..

[138] Hidalgo-Vila J., Martínez-Silvestre A., Pérez-Santigosa N., León-Vizcaíno L., Díaz-Paniagua C. (2020). High prevalence of diseases in two invasive populations of red-eared sliders (*Trachemys scripta elegans*) in southwestern Spain. Amphib. Reptil..

[139] Mihalca A.D. (2015). Ticks imported to Europe with exotic reptiles. Vet. Parasitol..

[140] Pietzsch M., Quest R., Hillyard P.D., Medlock J.M., Leach S. (2006). Importation of exotic ticks into the United Kingdom via the international trade in reptiles. Exp. Appl. Acarol..

[141] Nowak M. (2010). Parasitisation and localisation of ticks [*Acari: Ixodida*] on exotic reptiles imported into Poland. Ann. Agric. Environ. Med..

[142] Karesh W.B., Cook R.A., Bennett E.L., Newcomb J. (2005). Wildlife Trade and Global Disease Emergence. Emerg. Infect. Dis..

[143] Kar S., Rodriguez S.E., Akyildiz G., Cajimat M.N.B., Bircan R., Mears M.C., Bente D.A., Keles A.G. (2020). Crimean-Congo hemorrhagic fever virus in tortoises and Hyalomma aegyptium ticks in East Thrace, Turkey: Potential of a cryptic transmission cycle. Parasit. Vectors.

[144] Alexander D.J. (1995). The epidemiology and control of avian influenza and Newcastle disease. J. Comp. Pathol..

[145] UK Government Dangerous Dogs Act 1991. https://www.legislation.gov.uk/ukpga/1991/65/section/1/enacted.

[146] CITES Appendices. https://www.cites.org/eng/app/appendices.php.

[147] European Commission Single Market Scoreboard Performance per Member State. https://ec.europa.eu/internal_market/scoreboard/performance_by_member_state/index_en.htm.

[148] Billock J. Explore Extremes: Illegal in Iceland: Quirky Bans from the Land of Fire and Ice. https://www.smithsonianmag.com/travel/illegal-in-iceland-180957521/.

[149] Eur-lex Document 62007CA0219, OJ C 209. https://eur-lex.europa.eu/legal-content/EN/TXT/?uri=CELEX%3A62007CA0219.

[150] Di Silvestre I., van der Hoeven S. (2016). The Implementation of the Positive List for Mammal Pets in Belgium: A Success Story.

[151] Government of Flanders (2018). Flemish Animal Welfare Council—Opinion Guidance 28/11/2018. Positive List for Reptiles; Brussels. https://dierenwelzijn.vlaanderen.be/sites/default/files/atoms/files/Opinion%20guidance%20-%20Positive%20list%20for%20reptiles%2028112018_EN.pdf.

[152] Grand-Duché de Luxembourg (2018). Règlement Grand-Ducal du 16 Novembre 2018 Fixant les Listes Des Animaux Autorisés et les Modalités Particulières des Demandes D’autorisation de Détention. http://legilux.public.lu/eli/etat/leg/rgd/2018/11/16/a1055/jo.

[153] Government of Malta (2014). Subsidiary Legislation 439.16. Protection of Animals Offered in Pet Shops (Minimum Standards) Regulations. http://lawjournal.ghsl.org/viewer/280/download.pdf.

[154] Croatian Ministry of Environment Ordinance, OG 17/2017-404. https://narodne-novine.nn.hr/clanci/sluzbeni/2017_02_17_404.html.

[155] Lovdata Foundation Forskrift om Forbud Mot å Innføre, Omsette og Holde Eksotiske dyr. https://lovdata.no/dokument/SF/forskrift/2017-05-11-597.

[156] Lucca C.M. (2013). Keeping lions, tigers, and bears (Oh My!) in check: The state of exotic pet regulation in the wake of the Zanesville, Ohio massacre. Vill. Envtl. LJ..

[157] Drouet M. EXOTIC Pets Update (2013) Brief Summary of Exotic Pets Laws. https://www.animallaw.info/intro/exotic-pets-update-2013.

[158] Liebman M.G. Detailed Discussion of Exotic Pet Laws. https://www.animallaw.info/article/detailed-discussion-exotic-pet-laws.

[159] Michigan State University Map of Private Exotic PET Ownership Laws. https://www.animallaw.info/content/map-private-exotic-pet-ownership-laws.

[160] Madison Area Herpetological Society Wisconsin Reptile and AMPHIBIAN Ordinance Listing. http://www.madisonherps.org/kickstart/en/wisconsin-reptile-resources/education-articles/138-wisconsin-reptile-and-amphibian-ordinance-listing.

[161] Than K. Should the Ohio Exotic Animals Have Been Shot?. https://www.nationalgeographic.com/news/2011/10/111020-ohio-exotic-animals-shootings-thompson-farm-nation/.

[162] Ohio Legislature Ohio Exotic Animal Laws. http://uappeal.org/ohio.html.

[163] Nevada Legislature NAC: Chapter 503—Hunting, Fishing and Trapping; Miscellaneous Protective Measures. https://www.leg.state.nv.us/nac/nac-503.html.

[164] Idaho State Department of Agriculture Deleterious Exotic Animals. https://agri.idaho.gov/main/animals/deleterious-exotic-animals/.

[165] Nova Scotia Canada Wildlife as Pets: Importation & Captivity Requirements. https://novascotia.ca/natr/wildlife/laws/captivewildlife.asp.

[166] Wikipedia Lists of Municipalities in Canada. https://en.wikipedia.org/wiki/Lists_of_municipalities_in_Canada.

[167] CBC News Woman Mauled to Death by Tiger in B.C. Interior. https://www.cbc.ca/news/canada/british-columbia/woman-mauled-to-death-by-tiger-in-b-c-interior-1.635094.

[168] Province of British Columbia Controlled Alien Species. https://www2.gov.bc.ca/gov/content/environment/plants-animals-ecosystems/cas.

[169] Vaughan A. New Brunswick Introduces Exotic Animals act 4 Years after Boys’ Python Deaths. https://www.huffingtonpost.ca/2017/10/31/new-brunswick-introduces-exotic-animals-act-4-years-after-boys-python-deaths_a_23262321/.

[170] CanLII Exotic Animals Act, SNB 2017, c 52. https://www.canlii.org/en/nb/laws/stat/snb-2017-c-52/latest/snb-2017-c-52.html.

[171] Ison D.C. (2017). Mitigating nonresponse error in online surveys through research-based design and delivery. Surv. Pract..

[172] Convention on Biological Diversity. Precautionary Approach. https://www.cbd.int/marine/precautionary.shtml.

[173] Eur-Lex Document 12008E191. https://eur-lex.europa.eu/LexUriServ/LexUriServ.do?uri=CELEX:12008E191:EN:HTML.

[174] Vance Centre (2018). Global Pact Regional Review–African Union States. https://www.iucn.org/sites/dev/files/global_pact_regional_review_-_african_union_states.pdf.

[175] Patoka J., Magalhães A.L.B., Kouba A., Faulkes Z., Jerikho R., Vitule J.R.S. (2018). Invasive aquatic pets: Failed policies increase risks of harmful invasions. Biodivers. Conserv..

[176] Reaser J.K., Meyerson L.A., Von Holle B. (2008). Saving camels from straws: How propagule pressure-based prevention policies can reduce the risk of biological invasion. Biol. Invasions.

[177] Yanai Z., Dayan T., Mienis H.K., Gasith A. (2017). The pet and horticultural trades as introduction and dispersal agents of non-indigenous freshwater molluscs. Manag. Biol. Invasions.

[178] Chucholl C. (2013). Invaders for sale: Trade and determinants of introduction of ornamental freshwater crayfish. Biol. Invasions.

[179] FVE Regulation of Keeping Animals as Companion Animals through the Establishment of Lists; Federation of Veterinarians of Europe. https://www.fve.org/cms/wp-content/uploads/006_fve_position_on_positive_lists_of_exotic_species_final.pdf.

[180] De Briynea N., Iatridoua D. (2016). Challenges seen with treatment of exotic pets in veterinary practice. AWSELVA J..

[181] Stanford M. (2013). Keeping exotic pets. Vet. Rec..

[182] Kock R. (2014). Keeping exotic pets. Vet. Rec..

[183] Warwick C., Steedman C., Nicholas E. (2013). Veterinarian accountability and the exotic pet trade. AWSELVA J..

[184] The Pet Industry Joint Advisory Council (PIJAC) Canada (2013). Responsible Pet Ownership Review. http://clkapps.winnipeg.ca/DMIS/ViewPdf.asp?SectionId=335039.

[185] Colorado Division of Parks and Wildlife Chapter W-11—Wildlife Parks and Unregulated Wildlife. https://cpw.state.co.us/Documents/RulesRegs/Regulations/Ch11.pdf.

[186] Arkansas Game & Fish Commission Wildlife Pets. https://www.agfc.com/en/wildlife-management/captive-wildlife/wildlife-pets/.

[187] Flemish Government, Department of Environment Welke Reptielen Mag je Houden?. https://www.huisdierinfo.be/welke-reptielen-mag-je-houden.

[188] Warwick C., Frye F.L., Murphy J.B. (2013). Health and Welfare of Captive Reptiles.

[189] Burghardt G. (2013). Environmental enrichment and cognitive complexity in reptiles and amphibians: Concepts, review, and implications for captive populations. Appl. Anim. Behav. Sci..

[190] Warwick C., Arena P., Lindley S., Jessop M., Steedman C. (2013). Assessing reptile welfare using behavioural criteria. In Pract..

[191] EMODE Pet Score The First Step in Responsible Ownership. https://emodepetscore.com..

[192] Kiebler C.A., Bottichio L., Simmons L., Basler C., Klos R., Gurfield N., Roberts E., Kimura A., Lewis L.S., Bird K. (2020). Outbreak of human infections with uncommon Salmonella serotypes linked to pet bearded dragons, 2012–2014. Zoonoses Public Health.

[193] Bjelland A.M., Sandvik L.M., Skarstein M.M., Svendal L., Debenham J.J. (2020). Prevalence of Salmonella serovars isolated from reptiles in Norwegian zoos. Acta Vet. Scand..

[194] Bosch S., Tauxe R.V., Behravesh C.B. (2016). Turtle-associated salmonellosis, United States, 2006–2014. Emerg. Infect. Dis..

[195] Krag A. (2017). Personal communication.

[196] Warwick C., Arena P., Steedman C. (2009). Reptiles and Amphibians as Pets & the Norwegian Positive List Proposal. Assessment & Opinion. https://dyrevern.no/app/uploads/2019/11/herptil_rapport_2009.pdf.

[197] Norwegian Food Safety Authority (2015). Consultation Responses on the Draft Regulation Banning the Introduction, Sale and Keeping of Exotic Animals.

[198] Vanautryve E. Keeping of exotic animals in Belgium: The “positive list.”. Proceedings of the Import and Keeping of Exotic Animals in EU Auditorium.

[199] Van Tilburgh E. (2020). Personal communication.

[200] Government of Spain Documento consolidado BOE-A-2007-21490. https://www.boe.es/buscar/act.php?id=BOE-A-2007-21490.

[201] Birdlife International Will the EU’s New Invasive Species Regulation Miss the Chance to Save Billions of Euros?. https://www.birdlife.org/europe-and-central-asia/news/will-eus-new-invasive-species-law-miss-chance-save-billions-euros.

[202] Maceda-Veiga A., Escribano-Alacid J., Martínez-Silvestre A., Verdaguer I., Mac Nally R. (2019). What’s next? The release of exotic pets continues virtually unabated 7 years after enforcement of new legislation for managing invasive species. Biol. Invasions.

[203] Schuppli C.A., Fraser D. (2000). A framework for assessing the suitability of different species as companion animals. Animal Welfare..

[204] Koene P., de Mol R.M., Ipema B. (2016). Behavioral ecology of captive species: Using bibliographic information to assess pet suitability of mammal species. Front. Vet. Sci..

[205] Warwick C., Steedman C., Jessop M., Toland E., Lindley S. (2014). Assigning degrees of ease or difficulty for pet animal maintenance: The EMODE system concept. J. Agric. Environ. Ethics.

[206] Warwick C., Steedman C. Regulating pets using an objective positive list approach. JVEB.

[207] Fischer A., Bartsch F., Altherr S. (2015). Final Station: Living Room. Exotic Mammals as Pets.

[208] UNEP/Green Customs Initiative The Green Customs Guide to Multilateral Environmental Agreements. https://wedocs.unep.org/bitstream/handle/20.500.11822/25495/Green_Customs_MEAs.pdf?sequence=1&isAllowed=y.

[209] United Nations Office on Drugs and Crime (2020). World Wildlife Crime Report 2020.

[210] Siriwat P., Nijman V. (2020). Wildlife trade shifts from brick-and-mortar markets to virtual marketplaces: A case study of birds of prey trade in Thailand. J. Asia-Pac. Biodivers..

[211] Spee L.B., Hazel S.J., Dal Grande E., Boardman W.S.J., Chaber A.-L. (2019). Endangered exotic pets on social media in the Middle East: Presence and impact. Animals.

[212] CITES Investigating the Internet Wildlife Trade. https://www.cites.org/eng/news/world/19/7.php.

[213] Runhovde S.R. (2018). Illegal Online Trade in Reptiles from Madagascar 2018.

[214] Jensen T., Auliya M., Burgess N., Aust P., Pertoldi C., Strand J. (2018). Exploring the international trade in African snakes not listed on CITES: Highlighting the role of the internet and social media. Biodivers. Conserv..

[215] Eskew E., Ross N., Zambrana-Torrelio C., Karesh W. (2019). The CITES Trade Database is not a “global snapshot” of legal wildlife trade: Response to Can et al., 2019. Glob. Ecol. Conserv..

[216] Berkunsky I., Quillfeldt P., Brightsmith D.J., Abbud M.C., Aguilar J.M.R.E., Alemán-Zelaya U., Aramburú R.M., Arce Arias A., Balas McNab R., Balsby T.J.S. (2017). Current threats faced by Neotropical parrot populations. Biol. Conserv..

[217] Dee L.E., Karr K.A., Landesberg C.J., Thornhill D.J. (2019). Assessing vulnerability of fish in the U.S. marine aquarium trade. Front. Mar. Sci..

[218] Böhm M., Collen B., Baillie J.E., Bowles P., Chanson J., Cox N., Hammerson G., Hoffmann M., Livingstone S.R., Ram M. (2013). The conservation status of the world’s reptiles. Biol. Conserv..

[219] Frank E.G., Wilcove D.S. (2019). Long delays in banning trade in threatened species. Science.

[220] Lieberman S. CITES, the Treaty that Regulates Trade in International Wildlife, Is not the Answer to Preventing Another Zoonotic Pandemic. https://blogs.scientificamerican.com/observations/cites-the-treaty-that-regulates-trade-in-international-wildlife-is-not-the-answer-to-preventing-another-zoonotic-pandemic/.

[221] Vyawahare M. As Covid-19 Pandemic Deepens, Global Wildlife Treaty Faces Scrutiny. https://www.eco-business.com/news/as-covid-19-pandemic-deepens-global-wildlife-treaty-faces-scrutiny/.

