# Supplementary material for: Turning Negatives into Positives for Pet Trading and Keeping: A Review of Positive Lists
Source: Animals (Basel). 2020 Dec 10;10(12):2371. doi: 10.3390/ani10122371 (PMC7763047; doi:10.3390/ani10122371)
Supplement: Supplementary file 1 [file animals-10-02371-s001.pdf]

Questionnaire A

Can you assist our research?

You are invited to participate in a survey being conducted by animal welfare researchers in Europe, the USA and Canada. Key contacts are:

Europe:
Elaine Toland BSc(Hons) MRSB FRSPH (elaine@apa.org.uk) +44 (0)1273 674253

USA:
Dr Monica K. H. Bando, MS, BVSc, PhD, MRCVS (monicab@petaf.org) (509) 288-2821

Canada:
Michèle Hamers MSc, MRSB, EurProBiol (michelehamers@worldanimalprotection.ca) +1 416 369 0044 ext 115

The purpose of this study is to evaluate different approaches to regulating the trade and private ownership of wild animals as pets. Your participation in the survey will advance knowledge of 'best practice'. If you agree to participate in the study, you will be asked some straightforward questions. We estimate that it will take less than 5 minutes of your time to complete the questionnaire. You are free to contact the relevant investigator via the above email and phone number to discuss the survey.

After you complete the questionnaire, we may contact you with follow-up questions based on your answers and your willingness to provide your contact information. Your participation in this survey is voluntary. You may decline to answer any question and you have the right to withdraw from participation at any time.

Answers to the first survey question will be attributed to specific governments, but answers to all other questions will be aggregated and recorded as percentages. Email addresses will be kept during the data collection phase for tracking purposes only. A limited number of research team members will have access to personal data and this information will be stripped from the final dataset.

We have taken all reasonable measures to protect your identity and responses. The questions in this survey do not ask you to reveal any personally identifying information, the data are stored in a password protected database, and IP addresses are not collected by the researchers.

You can visit the SurveyMonkey privacy policy here.

Deadline for responses is 9th September 2020.

Do you agree to the above terms? By clicking Yes, you consent that you are willing to answer the questions in this survey.

- ☐ Yes
- ☐ No

What are 'positive' and 'negative' lists?

A positive list (‘approved’ list) permits the selling and private keeping of only those species that are: suitable to keep in the home in terms of animal welfare; and/or proportionately benign in terms of human health and safety; and/or sustainable in terms of relevant conservation status; and/or consistent with environmental preservation. All other species are by default prohibited from selling and private keeping, or may only be sold or kept with a special permit (e.g. by those who can demonstrate that they have specialist facilities or expertise).

By contrast, a negative list (‘banned’ list) prohibits the selling and private keeping of only those species that are: unsuitable to keep in the home in terms of animal welfare; and/or proportionately harmful in terms of human health and safety; and/or unsustainable in terms of relevant conservation status; and/or inconsistent with environmental preservation. Certain species may be permitted for selling and private keeping with a special permit (e.g. by those who can demonstrate that they have specialist facilities or expertise).

Please answer the question(s) below relating to positive lists:

1. Do you operate a Positive List of species that are permitted to be sold or privately kept as pets?

☐ Yes

☐ No

2. Are you developing a Positive List?

☐ Yes

☐ No

3. Are you considering introducing a Positive List?

☐ Yes

☐ No

4. Would you consider a Positive List?

☐ Yes

☐ No

QUESTIONNAIRE B

Can you assist our research?

You are invited to participate in a survey being conducted by animal welfare researchers in Europe, the USA and Canada. Key contacts are:

Europe: Elaine Toland BSc(Hons) MRSB FRSPH (elaine@apa.org.uk)
+44 (0)1273 674253
USA: Dr Monica K. H. Bando, MS, BVSc, PhD, MRCVS (monicab@petaf.org)
(509) 288-2821
Canada: Michèle Hamers MSc, MRSB, EurProBiol (michelehamers@worldanimalprotection.ca) +1 416 369 0044 ext 115

The purpose of this study is to evaluate different approaches to regulating the trade and private ownership of wild animals as pets. Your participation in the survey will advance knowledge of 'best practice'. According to our research, your government has a 'positive list' system (which lists animals that are permitted to be kept by private individuals rather than those that are prohibited) for regulating the trading and/or keeping of pets (see full definitions in the questionnaire). We are therefore very interested to learn about your observations and experience in this area. All responses will be anonymous and not attributed to any individual or government. Data will be collected in aggregate.

If you agree to participate in the study, you will be asked some straightforward questions. We estimate that it will take around 20 minutes of your time to complete the questionnaire. You are free to contact the relevant investigator via the above email and phone number to discuss the survey. Your participation in this survey is voluntary. You may decline to answer any question and you have the right to withdraw from participation at any time.

We have taken all reasonable measures to protect your identity and responses. The data are stored in a password protected database, and IP addresses are not collected by the researchers.

You can visit the SurveyMonkey privacy policy here.

Deadline for responses is 9th September 2020.

Do you agree to the above terms? By clicking Yes, you consent that you are willing to answer the questions in this survey.

- ☐ Yes
- ☐ No

What are 'positive' and 'negative' lists?

A positive list ('approved' list) permits the selling and private keeping of only those species that are: suitable to keep in the home in terms of animal welfare; and/or proportionately benign in terms of human health and safety; and/or sustainable in terms of relevant conservation status; and/or consistent with environmental preservation. All other species are by default prohibited from selling and private keeping, or may only be sold or kept with a special permit (e.g. by those who can demonstrate that they have specialist facilities or expertise).

By contrast, a negative list ('banned' list) prohibits the selling and private keeping of only those species that are: unsuitable to keep in the home in terms of animal welfare; and/or proportionately harmful in terms of human health and safety; and/or unsustainable in terms of relevant conservation status; and/or inconsistent with environmental preservation. Certain species may be permitted for selling and private keeping with a special permit (e.g. by those who can demonstrate that they have specialist facilities or expertise).

1. When was your Positive List implemented?

- ☐ Within the last year
- ☐ Within the last 5 years
- ☐ Within the last 10 years
- ☐ Over 10 years ago

2. Please describe the system or criteria that was used to formulate the Positive List?

3. What (if any) would you say were the main challenges in formulating the Positive List?

4. Do you consider the Positive List, as a means to regulate the exotic pet trade, to be an improvement on the previous system?

- ☐ Strongly agree
- ☐ Agree
- ☐ Undecided
- ☐ Disagree
- ☐ Strongly disagree

5. How would you describe the overall public response to the Positive List?

- ☐ Very supportive
- ☐ Moderately supportive
- ☐ Neutral
- ☐ Moderately unsupportive
- ☐ Very unsupportive

6. According to your own observations, what (if anything) could be improved with regard to the development and implementation of the Positive List?

7. Do you have anything further to add relevant to your experience of the Positive List system?

Telephone interview questions

PARTICIPANT INFORMATION SHEET

Study: *Review of Positive versus Negative Lists for pet trading and keeping*

Phase 2: Telephone interviews

European Author Contacts:

- Elaine Toland BSc(Hons) MRSB FRSPH
  Director, Animal Protection Agency, 15-17 Middle St, Brighton UK BN1 1AL
  (elaine@apa.org.uk)
  +44 (0)1273 674253

- Dr Albert Martinez-Silvestre DVM, PhD, Dipl ECZM
  Scientific Director, Catalonian Reptiles and Amphibians Rescue Centre, C/ Avinguda del Maresme, 45, Masquefa, 08783, Spain
  (crarc-masquefa@outlook.com)
  0034 619972497 / 0034 937726396

- Dr Vanessa Cadenas
  Zoological Inspector, Animal Protection, Biodiversity and Natural Environmental Section, Government of Catalonia, C/Anslem Clave, 1, 43004, Tarragona, Spain
  +34 977 21 65 62

Further to your participation in an online survey, you have been invited to take part in a telephone interview. Before you decide whether you would like to participate, please take the time to read the following information. Feel free to discuss it with the above authors if you wish. We will request your verbal consent prior to commencing the interview.

The purpose of the study is to evaluate different approaches to regulating the trade and private ownership of wild animals as pets in Europe, Canada and the USA. The work is being undertaken by scientists and vets associated with a number of animal welfare NGOs in Europe and North America. Your contribution via the interview will advance knowledge of 'best practice'.

You have been selected for interview because you confirmed in the online survey that either: you would consider or were considering a positive list of species that are permitted to be sold or privately kept as pets; or you were already in the process of developing a positive list. We estimate that it will take around 10 minutes to complete the interview. We don't anticipate that there are any risks associated with your participation.

Your participation is entirely voluntary. Any decision regarding participation will be confidential between you and the research team. You have the right to withdraw from participation at any time and any data collected from you would not be included in the study. You may decline to answer any question during the interview.
The interviewer will take notes but all information you give will be treated in the strictest confidence and will be fully anonymised. No individual or country data will be identifiable at any stage in the publication or presentation

of the findings. Data collected will be stored securely in a manner that is consistent with the General Data Protection Regulation.

If you have any further questions, please do not hesitate to contact the above authors.
Thank you for your time.

Telephone interview questions

- How would you describe your role in the department with specific regard to regulation of exotic pet trade and keeping?
- What would you say has prompted the government to consider/develop a positive list?
- What current problems do you experience with regard to exotic pet trading and keeping?
- How do you envisage positive lists working in terms of addressing current problems?
- Are there any challenges you face or obstacles that you anticipate in introducing a positive list?
- Do you have anything else you'd like to add that you think may be of interest?
